# Partitioning photoperiod- and chilling-driven cold hardiness in a *Vitis riparia* × *V. vinifera* F_1_ population

**DOI:** 10.3389/fpls.2026.1916819

**Published:** 2026-09-30

**Authors:** Hava Delavar, Ozkan Kaya, Summaira Riaz, Harlene Hatterman-Valenti

**Affiliations:** 1 Department of Plant Sciences, North Dakota State University, Fargo, ND, United States; 2 Department of Life Sciences, Western Caspian University, Baku, Azerbaijan; 3 Erzincan Horticultural Research Institute, Republic of Turkey Ministry of Agriculture and Forestry, Erzincan, Türkiye; 4 Department of Horticulture and Agronomy, Faculty of Agriculture, Kyrgyz-Turkish Manas University, Bishkek, Kyrgyzstan; 5 US Department of Agriculture, Agricultural Research Service, Crop Diseases, Parlier, CA, United States

**Keywords:** differential thermal analysis, dormancy, genotype × environment interaction, low-temperature exotherm, *Vitis*

## Abstract

Grapevine cold hardiness governs cultivar distribution and winter survival, and is a key constraint on viticulture in cold regions. We evaluated bud cold hardiness in a *Vitis riparia* × *Vitis vinifera* F_1_ population by differential thermal analysis across three environments (a California vineyard, an NDSU field, and an NDSU greenhouse), sampled nine times through the 2023–2024 dormant season. Mean low-temperature exotherms (LTEs) differed significantly among sampling times, and hierarchical clustering separated the genotypes into five hardiness classes defined by mean LTE, from very hardy (≤ −21.4 °C) to very susceptible (≥ −13.4 °C). Field vines reached a midwinter maximum of −22.7 °C, whereas greenhouse vines were most susceptible (−14.6 °C), and nine genotypes were hardier than the tender parent. Genotype accounted for only 7% of the phenotypic variance, giving a low single-observation heritability (H²i = 0.07) but a moderate entry-mean heritability (H²c = 0.49), which are distinct statistics reported separately. The seasonal trajectory was well described by logistic and response-surface models (R² = 0.99) and, on an accumulated-chilling axis, by a Weibull model (half-hardening at 184 chilling degree-days). A state-space model estimated the chilling contribution at ≈6.7 °C and identified three genotypes (536-078, 539-127, 536-142) that attained most of their hardiness under the short-day, moderate-cooling greenhouse regime without sub-zero exposure; because that regime combined shortening photoperiod with a 4 °C decline, these are described as short-day/low-temperature responsive rather than photoperiod-responsive. Mean hardiness and plasticity were uncorrelated, providing no evidence of a hardiness–stability trade-off. The December field and greenhouse samplings exposed the greatest among-genotype variance and are the most informative conditions for future QTL mapping; the nine genotypes hardier than the tender parent, and a hardy, stable subset, are identified as parents for cold-hardiness introgression.

## 1 Introduction

The expansion of viticulture into cool- and cold-climate regions is constrained primarily by extreme low temperatures (Delavar et al., 2026). When these temperatures exceed the tolerance of the vine, they injure buds, canes, cordons, and trunks, and the resulting damage not only reduces yield but also raises production costs through the retraining and replacement of affected vines (Zhang et al., 2012; Kaya and Köse, 2017; Zabadal et al., 2007). Of the tissues at risk, the dormant bud is the most consequential: although it attains its greatest hardiness in midwinter, it is then that it faces the lowest temperatures of the year, so freeze injury to the dormant bud rather than to the deacclimated early-spring bud is the principal limiting factor for grapevine cultivation outside Mediterranean climates (Schnabel and Wample, 1987). The severity of this injury, however, is not fixed: cold hardiness varies markedly among *Vitis vinifera* cultivars and fluctuates seasonally, such that sensitive cultivars may tolerate only −10 °C in early winter, whereas hardy cultivars survive to −26 °C once fully acclimated (Mills et al., 2006; Ferguson et al., 2011). Greater hardiness still is found beyond the cultivated species, as wild *Vitis* species and their interspecific hybrids generally outperform *V. vinifera* (Londo and Kovaleski, 2017), with some enduring midwinter temperatures of −35 to −40 °C (Pierquet and Stushnoff, 1980; Andrews et al., 1984). Foremost among these wild sources is the North American riverbank grape *Vitis riparia*, whose exceptional cold hardiness and extensive geographic distribution have made it a favored donor of hardiness in breeding programs (Garris et al., 2009).

Building on this resource, the dominant breeding strategy hybridizes high-quality *V. vinifera* with wild germplasm in order to combine superior fruit quality with abiotic-stress tolerance, and in particular the cold hardiness contributed by *V. riparia* (Fennell, 2004; Carbonell-Bejerano et al., 2015; Ferrandino and Lovisolo, 2014). Pursued over several decades, this strategy has produced cold-hardy “northern hybrids” adapted to the cool, humid climates of the eastern and northcentral United States (Bordelon, 2001; Hemstad and Luby, 2002; Reisch et al., 2002; Köycü et al., 2017; Delavar et al., 2026), as well as comparably hardy selections developed in Canada, the former Soviet Union, France, and Germany, several of which tolerate −31 to −32 °C (Fisher and Jamieson, 1998; Nenko et al., 2019; Bordelon et al., 1997; Liu et al., 2004). Despite this sustained progress, however, the genetic dissection of cold hardiness in such crosses remains incomplete. The central obstacle is that the trait is governed jointly by genotype and a strongly fluctuating environment, so that the phenotype expressed in any single winter is an imperfect guide to the genetic potential that a breeder ultimately seeks to select.

This environmental dependence, while it complicates selection, is itself mechanistically well understood. Because maximum cold hardiness is not attained consistently across winters but depends on specific environmental cues, principally photoperiod and temperature (Londo and Kovaleski, 2017), its expression is best viewed as a seasonal process rather than a fixed value. In that process, short days and low temperatures together trigger growth cessation, dormancy, and the progressive acquisition of hardiness during acclimation, which is subsequently reversed during deacclimation and reacclimation (Fennell and Hoover, 1991; Kalberer et al., 2006; Xiao et al., 2008). Underlying these transitions are the accumulation of cryoprotective solutes, changes in membrane composition, and the induction of cold-responsive genes (Xiao et al., 2008). What matters most for breeding, however, is that genotypes differ in which cues they require: short days induce growth cessation and dormancy and act synergistically with declining temperature to enhance bud cold acclimation, but acclimation attributable to short days alone, in the absence of low night temperatures, has not been demonstrated in grapevine. Schnabel and Wample (1987) explicitly concluded that no acclimation could be attributed to a 10.5-h photoperiod before the onset of low night temperatures, and Wake and Fennell (2000) examined growth cessation and bud dormancy under short photoperiod rather than bud cold acclimation. Genotypes may nevertheless differ in the relative weight they place on the two cues, some acclimating substantially under short days combined with only moderate cooling, and others requiring sustained sub-zero exposure (Schnabel and Wample, 1987; Fennell and Hoover, 1991; Wake and Fennell, 2000). This difference is consequential, because a photoperiod-driven component of hardiness is comparatively stable across years and locations, whereas a chilling-driven component varies with the severity of the winter, and the two therefore carry very different implications for both mapping and cultivar deployment. Yet despite the extensive descriptive work summarized above, these two components have, to our knowledge, not been partitioned quantitatively within a single segregating *Vitis* population. The existing work is either descriptive or restricted to a small number of established cultivars: Schnabel and Wample (1987) manipulated photoperiod and temperature factorially but in a single *V. vinifera* cultivar, so that no genetic variance could be estimated; Fennell and Hoover (1991) and Wake and Fennell (2000) compared a small number of *Vitis* genotypes under short photoperiod but quantified growth cessation and dormancy rather than partitioning the two acclimation drivers; Garris et al. (2009) mapped photoperiod-induced growth cessation in *V. riparia* but did not measure bud cold hardiness; and Londo and Kovaleski (2017) and Ferguson et al. (2011) characterized or modelled hardiness across germplasm and across thermal time, respectively, without separating the photoperiod and chilling contributions within a segregating family. None of this work therefore yields a genotype-resolved estimate of the two components in a mapping population; the broad-sense heritability of the trait is seldom reported for grapevine families of this kind; and the sampling conditions that best expose genetic variation among genotypes, and therefore offer the greatest power for selection, have not been formally identified.

To address these gaps simultaneously, and to the best of our knowledge for the first time, we combined a common F_1_ mapping population, replicated sampling across contrasting environments, and an explicit quantitative framework. Specifically, we evaluated cold hardiness in this F_1_ population across three environments that differ in their photoperiod and chilling regimes. These three environments were chosen deliberately so as to span a gradient in the two cues described above. The NDSU field combines a natural shortening photoperiod with progressive natural chilling well below zero; the California vineyard combines a natural shortening photoperiod with a mild winter and limited sub-zero exposure; and the NDSU greenhouse imposes a controlled shortening photoperiod together with a modest, controlled decline in temperature that does not extend below 4 °C. The three environments therefore differ in both cues simultaneously, and the contrast they support is set out in Section 2.2. Within this design, we asked four questions, of which (1) and (2) are the primary objectives of the study and (3) and (4) are secondary, exploratory objectives derived from them: (1) how is the variance in cold hardiness partitioned among genotype, environment, and their interaction, and what is the broad-sense heritability of the trait?; (2) what mathematical form best describes the acclimation trajectory, and can the photoperiod- and chilling-driven contributions be separated and quantified?; (3) which genotypes acquire hardiness without sub-zero exposure, and are these the same genotypes that prove most resilient to midwinter warm spells?; and (4) which sampling environment and time expose the greatest genetic variation among genotypes, and therefore provide the most informative conditions for phenotyping and selection for cold hardiness in grapevine?

## 2 Materials and methods

### 2.1 Plant material and propagation

This study was conducted during the 2023–2024 dormant season on an F_1_ population derived from a controlled cross between a cold-hardy female parent (*Vitis riparia* PI588258) and a *V. vinifera* pollen donor, ‘Fresno Seedless’. ‘Fresno Seedless’, a sibling of ‘Flame Seedless’, was developed from the cross [(‘Cardinal’ × ‘Thompson Seedless’) × ((‘Red Malaga’ × ‘Tifafihi Ahmer’) × (‘Muscat of Alexandria’ × ‘Thompson Seedless’))] made in 1961 at the U.S. Horticultural Field Station, Fresno, California. The *V. riparia* × ‘Fresno Seedless’ cross was made in 2014, and the resulting F_1_ population was established in 2015 at the USDA-ARS San Joaquin Valley Agricultural Sciences Center, Parlier, California (36.5962° N, 119.5123° W; 36°35′46″ N, 119°30′44″ W), with 3.66 m between rows and 0.91 m between vines. Vines were grown on their own roots, spur-pruned to 8–10 buds, and trained to a single T-trellis.

Hardwood cuttings for propagation were collected at Parlier during the first week of January 2023, sealed in polyethylene bags to prevent desiccation, and shipped overnight to North Dakota State University (NDSU). On arrival, cuttings were held at 4 °C for one month to satisfy the chilling requirement, then inspected visually and by hand for adequate moisture and the absence of mold, each bundle being examined for surface mold growth, softening or discoloration of the bark, and desiccation of the cut ends. Dry cuttings were submerged in warm water (≈ 21–29 °C) for 6–12 h; where mold was present, it was removed by spraying with water followed by a 30-min soak in 10% bleach and a tap-water rinse. For callusing, cuttings were arranged over a 2.5-cm layer of moist callus mix (equal parts vermiculite and perlite) in a covered box, overlaid with moist newspaper, and held in a callus room at 26–29 °C under high relative humidity (approximately 85–95% RH); moisture was checked daily and callus and root initiation assessed after 10–14 days. Rooted cuttings were waxed at the bud end with grafting wax (≈ 77 °C), inserted into plant bands filled with moistened rooting mix (equal parts peat and perlite), and returned to the callus room (≈ 27–29 °C, near-100% relative humidity). Bands were then moved to a greenhouse with bottom heat (≈ 27–29 °C), ambient temperature of ≈ 24–29 °C, at least 50% shade, and elevated humidity (**Figure 1**). Once roots emerged and green shoots appeared, plants were hardened off under continued shade for several days. After approximately one month, shoots were cut back to two buds to promote vigorous development, and the plants were divided into two groups for field and greenhouse establishment (**Figure 1**).

**Figure 1:**
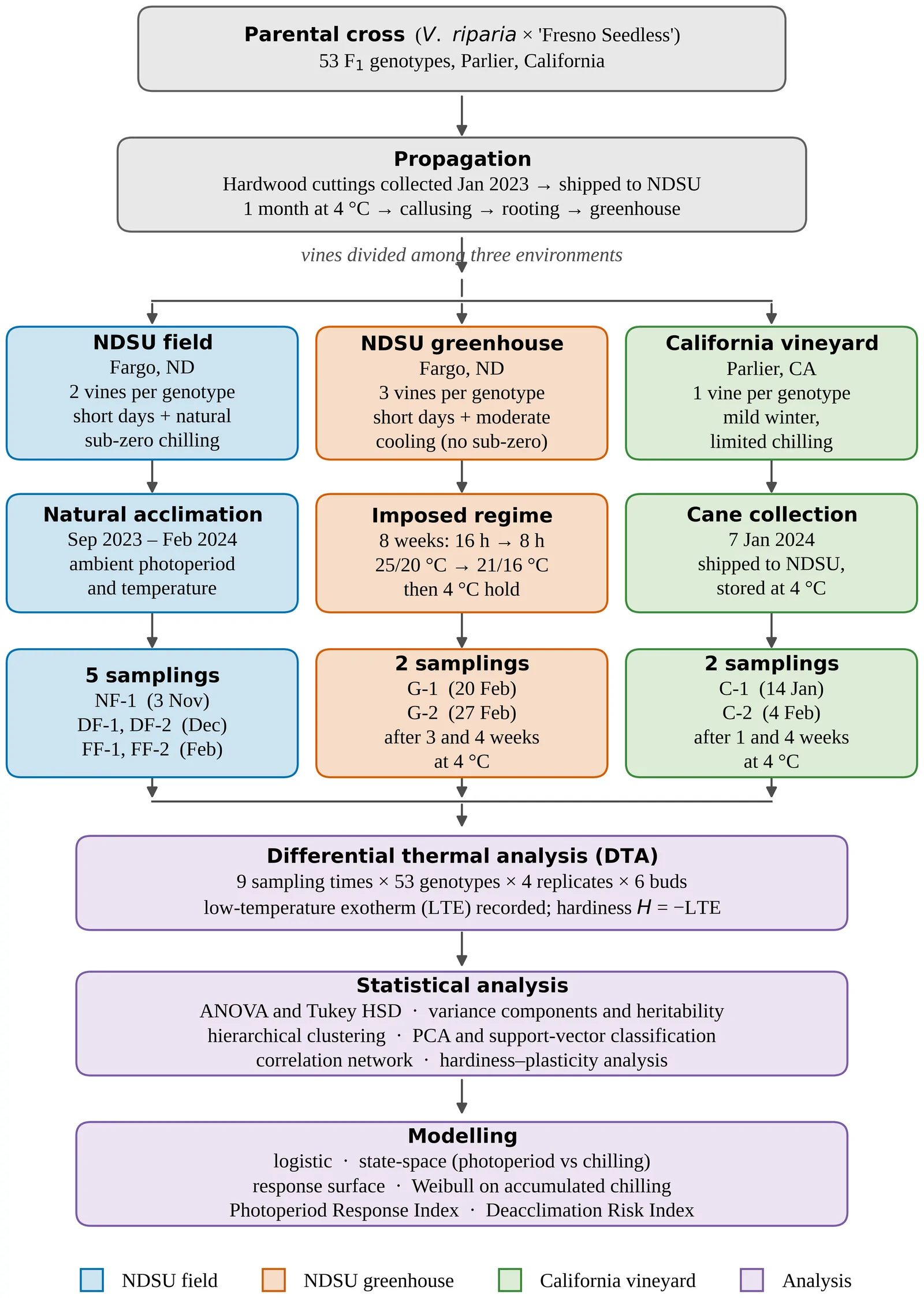
Experimental workflow. Propagation, environmental treatments, sampling scheme and analytical pipeline for the 53 F_1_ genotypes. Sampling codes and calendar dates are given in **Supplementary Table S1**; the greenhouse regime is detailed in **Supplementary Table S3**.

To avoid ambiguity, the chronology of the experiment was as follows. Hardwood cuttings for propagation were collected at Parlier during the first week of January 2023 and shipped to NDSU; callusing, rooting and greenhouse establishment took place between February and June 2023; the rooted vines were transplanted to the NDSU field and greenhouse in June 2023 and fertilized on 17 July 2023; the field and greenhouse vines were then sampled for DTA through the 2023–2024 dormant season (November 2023 to February 2024).

Separately, and for the California environment only, one-year-old canes were collected from the original F_1_ vines standing in the Parlier vineyard in the first week of January 2024 (Section 2.3). The January 2023 collection therefore supplied the propagation material, whereas the January 2024 collection supplied the California thermal analysis (DTA) samples; these are distinct events. The exact calendar dates of every sampling are given in **Supplementary Table S1**.

### 2.2 Experimental design and the three growing environments

Cold hardiness was evaluated in the same F_1_ genotypes across three contrasting environments, chosen so that the photoperiod- and chilling-driven components of acclimation could be separated. The California vineyard (Parlier) represents a mild-winter field environment in which vines experience a natural shortening photoperiod but limited sub-zero exposure. The NDSU field (Fargo) represents a cold-winter environment combining a natural shortening photoperiod with progressive natural chilling. The NDSU greenhouse (Fargo) represents a controlled environment in which photoperiod and temperature were imposed independently of natural weather, combining a shortening photoperiod with a limited decline in temperature and, critically, with no exposure below 4 °C. We emphasize that the three environments are not orthogonal contrasts of photoperiod and temperature: all three differ in both cues simultaneously, and the greenhouse regime is therefore best described as a short-day/moderate-cooling treatment rather than as a photoperiod-only treatment. The design consequently supports the estimation of the additional hardiness conferred by sustained sub-zero chilling, over and above a short-day/moderate-cooling regime, but it does not permit a factorial decomposition of photoperiod and temperature effects. A fully orthogonal design, in which vines receive a common photoperiod under contrasting temperature regimes and a common temperature under contrasting photoperiods, would be required for that decomposition, and is identified as a priority for future work (Section 4.6).

The NDSU field planting was established at the NDSU Campus Agricultural Research Center, Fargo (46.89° N, 96.81° W; 46°53′24″ N, 96°48′36″ W) in a randomized complete block design with two replicates, at 2 m between rows and 0.5 m within rows. One-year-old vines were trained to a single trunk on a bamboo stake. Each vine received drip irrigation (3.79 L h^-^¹ emitter, 30 min each morning, with supplemental irrigation as required), and ≈ 100 g of slow-release fertilizer (14-14-16; Multicote 4, Haifa North America, Savannah, GA) was incorporated into the top 10 cm of medium on 17 July 2023. Each genotype was represented by two vines in the NDSU field, one in each of the two randomized complete blocks. At each field sampling, one cane was collected from each of the two vines per genotype, so that the buds analyzed for a given genotype at a given sampling time derived from both vines rather than from a single vine; the four DTA replicates of six buds per genotype and sampling time (Section 2.4) were therefore distributed across the two vines, two replicates per vine. In the greenhouse, each genotype was represented by three vines, one in each of the three blocks; canes were collected from all three vines, and the four DTA replicates were distributed across those three vines. In the California vineyard, each F_1_ genotype is represented by a single vine at Parlier, and the four DTA replicates of six buds per genotype and sampling time were drawn from separate canes of that vine; the California replication is therefore within-vine (pseudo-replication at the vine level), and the variance components estimated for that environment must be interpreted accordingly. The number of vines per genotype was constrained by the size of the mapping population and the quantity of propagation material available, and replication was maximized at the level of buds and canes within each vine; the resulting estimates of genotypic effects should therefore be read as provisional and in need of confirmation in a larger planting. This limitation is revisited in Section 4.6.

Ambient conditions at the two outdoor sites were characterized so that the temperature and light environment experienced during acclimation can be reconstructed by the reader. For Fargo, daily maximum, minimum and mean air temperature and daily total solar radiation were obtained from the North Dakota Agricultural Weather Network (NDAWN) Fargo station (station no. 23; 46.89° N), with temperature converted from °F to °C and solar radiation from langleys to MJ m^-^² d^-^¹. For Parlier, station records from the California Irrigation Management Information System (CIMIS) were not accessible, and the corresponding daily values were instead obtained from the ERA5/ERA5-Land reanalysis (Open-Meteo Historical Weather API) for the grid cell containing the University of California Kearney Research and Extension Center, Parlier (36.59° N, 119.45° W, 104 m); these are modelled reanalysis values rather than station observations and are interpreted accordingly. Values for the period from 1 September 2023 to 29 February 2024 are presented in **Supplementary Table S2** and **Supplementary Figure S1**. Day length at each site and date was computed astronomically from latitude and day of year and is a calculated rather than a measured quantity. Conditions in the greenhouse were imposed rather than observed, and the photoperiod and temperature regime applied there is given in **Supplementary Table S3**.

The North Dakota State University (NDSU) greenhouse planting was established at the NDSU Campus Agricultural Research Greenhouse, Fargo, in a randomized complete block design with three replicates, under unshaded glass at 25 °C/20 °C and 40%/70% relative humidity (day/night). Supplemental high-pressure sodium lighting (Sunlight Supply, Woodland, WA) maintained a 16-h photoperiod whenever photosynthetic photon flux density fell below 600 µmol m^-^² s^-^¹. Plants were watered to container capacity each morning (approximately 400 mL per pot, adjusted to demand) and fertilized every other day with 100 ppm N water-soluble fertilizer (20-20-20; Peters Professional, Marysville, OH). Uniform one-year-old vines with five to six leaves were trained to a single trunk. Once vines reached 12 nodes, a combined short-day and cooling treatment was imposed. The treatment began from the pre-treatment conditions of a 16-h photoperiod and 25 °C day/20 °C night. Over the following eight weeks the photoperiod was shortened by 1 h per week, from 16 h to 8 h, and the day temperature was lowered by 2 °C in alternate weeks, from 25 °C to 21 °C, with the night temperature lowered in parallel from 20 °C to 16 °C, so that the regime ended at an 8-h/16-h (day/night) photoperiod and a temperature 4 °C below the starting point (21 °C day/16 °C night). The complete week-by-week schedule is given in **Supplementary Table S3**. At the end of week 8 the vines were transferred to a 4 °C chamber under an 8-h/16-h photoperiod; differential thermal analysis (DTA) was performed after three weeks (first DTA, G-1) and four weeks (second DTA, G-2) at 4 °C. The greenhouse regime therefore combined a shortening photoperiod with a moderate temperature decline and a subsequent constant 4 °C hold, and at no point included exposure below 4 °C; it does not constitute a photoperiod-only treatment, and is referred to throughout as the short-day/moderate-cooling regime.

### 2.3 Cane collection and sampling times

Sampling was designed to capture the full acclimation trajectory in the field while bracketing the short-day/moderate-cooling response in the greenhouse. The calendar dates of all nine samplings are listed in **Supplementary Table S1**; in the text the samplings are referred to by week for readability. In the California vineyard, one-year-old canes were collected during the first week of January 2024, selecting shoots with dark-brown, well-developed periderm on eight or more internodes (8.1–10.0 mm diameter), two hours after sunrise to minimize diurnal variation. Canes were bagged, shipped overnight to NDSU, and held at 4 °C for one week, after which one group was analyzed immediately (C-1) and a second group was held at 4 °C for an additional three weeks before analysis (C-2). At the NDSU field, canes were collected in the first week of November (NF-1), the first and third weeks of December (DF-1, DF-2), and the first and third weeks of February (FF-1, FF-2), with one cutting taken per replicate per vine. The sampling intensity was constrained by the number of vines available per genotype (Section 2.2); to compensate, each cutting yielded six buds and the four DTA replicates were pooled across these, so that each genotype-by-time hardiness estimate rests on 24 buds. The consequent limitation for the estimation of genotypic effects is acknowledged and discussed in Section 4.6. In the NDSU greenhouse, the first DTA (G-1) followed three weeks and the second (G-2) four weeks of cold acclimation, as described above. Each cane comprised eight or nine nodes, and cold hardiness was determined on dormant buds at the 3^rd^–8^th^ nodes.

An important qualification applies to the California samples. Because the canes had to be shipped from Parlier to Fargo, the C-1 buds were analyzed after one week at 4 °C and the C-2 buds after a further three weeks at 4 °C, whereas the NDSU field buds were analyzed within 24 h of collection. The California values therefore do not represent field hardiness at the moment of collection: they represent the hardiness of Parlier-grown buds after a defined period of post-collection storage at 4 °C, and C-2 in particular incorporates four weeks of additional artificial chilling. Storage at 4 °C is known to alter measured hardiness in grapevine buds (Kose et al., 2026), and the C-1 to C-2 increase in hardiness (−16.5 to −17.9 °C) is consistent with continued acclimation in storage rather than with acclimation in the Parlier vineyard. The comparison between California and the other environments is thus confounded by storage duration, and the “California vineyard” values should be read as “Parlier-grown, cold-stored” values throughout. We retain them because they extend the chilling gradient, but we do not interpret them as an unbiased estimate of field acclimation in a mild-winter climate, and Section 4.6 sets out the implications of this confound for the conclusions drawn.

### 2.4 Evaluation of bud cold hardiness by DTA

Bud cold hardiness was assessed by DTA following Mills et al. (2006) and Kaya and Köse (2020). Buds from the 3rd to the 8th node were excised with ≈ 2 mm of surrounding tissue and arranged on thermoelectric module (TEM) trays within a Tenney Junior environmental test chamber (model T2C-A-F4T, Thermal Product Solutions, New Columbia, PA) controlled by a TestEquity controller (Moorpark, CA). The protocol comprised a 1-h equilibration at 4 °C followed by cooling from 4 °C to −44 °C at 4 °C h^-^¹. The voltage output (mV) of each TEM was recorded continuously, and the low-temperature exotherm (LTE) of each bud, the temperature at which lethal intracellular freezing occurs (Sakai and Larcher, 1987), was identified from the resulting peaks. The design comprised four replicates of six buds, giving 24 buds per genotype at each sampling time, from which the mean lethal temperature (LTE_50_) was determined as a quantitative measure of bud cold hardiness.

### 2.5 Statistical analysis and modeling

All LTE values are reported as mean ± standard error (SE). For all analyses, cold hardiness (*H*) was expressed as *H* = −LTE, so that higher values denote greater hardiness. Analyses were performed in R 4.3.1 (R Core Team, 2023) and Python 3.11. The ANOVA, Tukey HSD tests, variance-component estimation and correlation network were carried out in R (packages lme4, with figures drawn using ggplot2; Wickham, 2016, emmeans and igraph); the model fitting, principal component analysis, support-vector classification and bootstrap procedures were carried out in Python, using NumPy and SciPy for the curve fitting, scikit-learn for the PCA and support-vector machine, and statsmodels for the response-surface regression. Differences in LTE among the nine sampling times and among the three environments were tested by analysis of variance (ANOVA), with means separated by Tukey’s honestly significant difference (HSD) test at *p* < 0.05 (package agricolae; Mendiburu, 2023); sampling times were assigned compact-letter-display groups accordingly. Variance components were estimated by restricted maximum likelihood from a two-way random-effects model fitted to the individual replicate LTE observations (four replicates of six buds per genotype per sampling time), with genotype, environment, and their interaction as random terms and the residual capturing within-environment replicate and sampling-time variation. Because the model was fitted to individual replicate values rather than to genotype means, the residual term is large relative to the genotypic term. Two heritability statistics, which answer different questions, were therefore computed and are reported separately. The single-observation broad-sense heritability (*H)*², *i*) was calculated as σ²G/(σ²G + σ²GE + σ²e) and equals 0.07. The entry-mean (broad-sense) heritability (*H*², *c*), which describes the reliability of a genotype mean averaged over the *e* = 3 environments and *r* = 4 replicates used here, was calculated following Falconer and Mackay (1996) as σ²G/(σ²G + σ²GE/*e* + σ²e/*re*), and equals 0.49. The value of 0.49 is therefore not the proportion of phenotypic variance explained by genotype; it is the squared correlation between the genotype means observed here and their true genotypic values, and it is specific to this number of environments and replicates. Both statistics are reported in **Figure 2A** and in the text, and all statements about selectable genetic variation are referred explicitly to one or the other. Relationships among the nine sampling times were summarized by a Pearson correlation matrix and visualized as a correlation network (edges drawn for |*r*| > 0.5). Genotypes were clustered by their LTE profiles using Ward’s hierarchical clustering and displayed as a dendrogram-linked heatmap (package pheatmap; Kolde, 2019). Multivariate structure was further examined by principal component analysis (PCA) of the standardized LTE profiles, and a radial-basis-function support-vector machine was trained on the first two principal components to delineate cold-hardiness classes.

**Figure 2:**
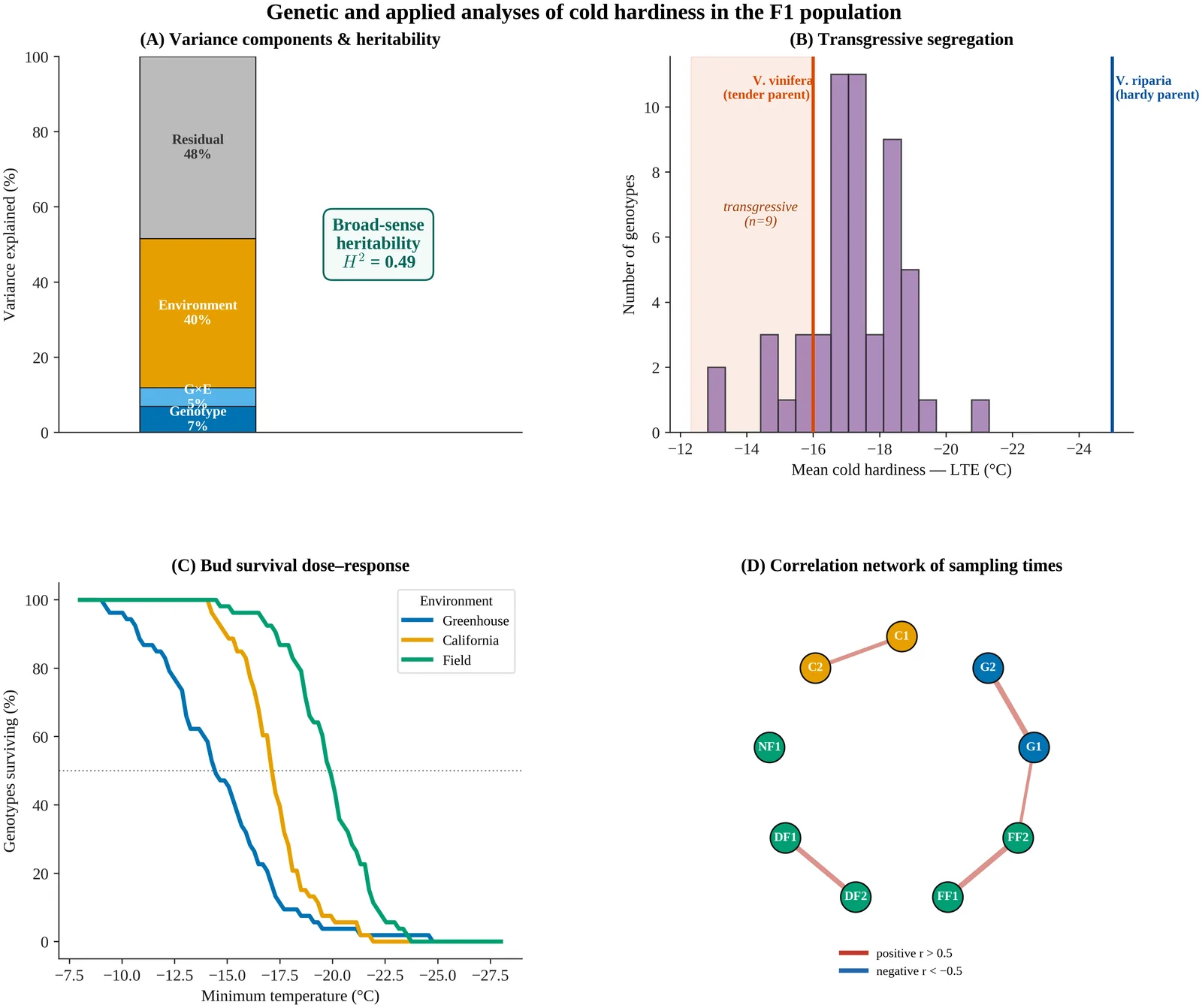
Genetic and applied analyses of cold hardiness in the F1 population. **(A)** Partitioning of phenotypic variance into genotype (7%), genotype × environment (5%), environment (40%) and residual (48%) components; the value shown in the panel as "broad-sense heritability, H^2^ = 0.49" is the entry-mean heritability (H^2^c) for genotype means taken over three environments and four replicates, whereas the corresponding single-observation broad-sense heritability is H^2^i = 0.07 (Section 2.5). **(B)** Distribution of mean cold hardiness across the 53 genotypes, with the tender (*V. vinifera*) and hardy (*V. riparia*) parental means marked; the nine genotypes annotated in the panel exceed the tender parent but none exceeds the hardy parent, and this is therefore segregation toward the hardier parent rather than transgressive segregation in the strict sense. **(C)** Proportion of genotypes surviving as a function of minimum temperature in each of the three environments. **(D)** Correlation network of the nine sampling times (edges drawn for |r| > 0.5).

The seasonal acclimation trajectory was characterized using four complementary models. These approaches follow established practice in dormancy and cold-acclimation modeling: logistic and Weibull functions have been used to describe hardiness and chilling responses in grapevine and other woody perennials (Ferguson et al., 2011; Kovaleski et al., 2018), and state-space or thermal-time formulations of acclimation and deacclimation have been developed for dormant buds by Kovaleski et al. (2018) and Ferguson et al. (2014). (i) A logistic hardening function, LTE(*t*) = *L*_max_ + (*L*_min_ − *L*_max_)/(1 + *e*^k(t−t0)^), was fitted to the field time course to estimate the asymptotic hardiness (*L*max), the deacclimated baseline (*L*min), the hardening rate (*k*), and the inflection point (*t*_0_). (ii) A mechanistic state-space model, dH/dt = a_P·φ(t)·(*H*max − *H*) + a_C·χ(t)·(*H*max − *H*) − d·(*H* − *H*min), was jointly calibrated to the field and greenhouse data to partition the photoperiod gain (a_P) and chilling gain (a_C). The two driving signals were defined explicitly as follows. The photoperiod signal φ(t) was defined as the normalized shortfall of day length below a 14-h threshold, φ(t) = min{1, max[0, (14 − D(t))/6]}, where D(t) is the astronomical day length in hours at the site latitude and date, so that φ = 0 at day lengths of 14 h or more and φ = 1 at a day length of 8 h; for the greenhouse, D(t) was the imposed photoperiod of the corresponding treatment week (**Supplementary Table S3**) rather than the astronomical value. The chilling signal χ(t) was defined as the normalized daily chilling accumulation, χ(t) = min{1, max[0, (5 − T̄(t))/15]}, where T̄(t) is the daily mean air temperature (°C) at the site, so that χ = 0 at daily means of 5 °C or above and χ = 1 at a daily mean of −10 °C or below; for the greenhouse, T̄(t) was the imposed treatment temperature, giving χ = 0 throughout the eight-week ramp and χ = 0.067 during the 4 °C hold. Both signals were computed at daily resolution from the weather-station and treatment records described in Section 2.2 and averaged over each inter-sampling interval before fitting. H_max and H_min were fixed at 27 and 13 °C respectively, corresponding to the extreme observed hardiness values, and a_P, a_C and d were estimated. The base temperature of 5 °C used for χ(t) and for the chilling degree-day axis (below) was adopted for consistency with the thermal-time model of Ferguson et al. (2011) for dormant grapevine buds; because this choice is not derived from first principles, a sensitivity analysis over base temperatures of 0, 5 and 10 °C is reported in **Supplementary Table S4**. The ranking of genotypes by hardening kinetics was essentially unaffected (Spearman ρ = 0.97 for genotype-level τ_50_ at both the 0 °C and 10 °C bases against the 5 °C reference), and the estimated contribution of sustained sub-zero chilling remained within 6.7 to 7.9 °C across the three bases, so the conclusions drawn below do not depend on this choice. (iii) A quadratic response-surface model, *H* = β_0_ + β_1_t + β_2_c + β_11_t² + β_22_c² + β_12_tc, described hardiness as a function of hardening time (*t*) and chilling intensity (*c*). (iv) A Weibull function, *H(τ) = H_min_ + (H_max_ − H_min_)[1 − e^−(τ/λ)κ^]*, was fitted on an accumulated-chilling axis (τ, degree-days, base 5 °C) to estimate the scale (λ) and shape (κ) parameters and the half-hardening point (τ_50_). Model fit was assessed by the coefficient of determination (R²). Finally, a Photoperiod Response Index (PRI = (*H*_GH − *H*_0_)/(*H*_Field − *H*_0_)) was computed for each genotype to quantify the fraction of its maximum (February field) hardiness that was attained under the short-day/moderate-cooling greenhouse regime, that is, in the absence of sub-zero exposure; H_0_ was the deacclimated baseline of 13 °C. The PRI is an index of the response to the greenhouse regime as a whole and not of the response to photoperiod in isolation, for the reasons set out in Section 2.2. The Deacclimation Risk Index (DRI) was defined as the fraction of a genotype’s peak field hardiness that was attributable to the chilling term of the state-space model, DRI = (H_field − H_GH)/(H_field − H_0_), and hence the fraction predicted to be lost when the chilling signal is withdrawn. The midwinter warm spell was simulated by setting χ(t) = 0 and holding φ(t) at its 1 February value for a continuous 10-day period beginning on 1 February, with the daily mean temperature raised to 10 °C, and then integrating the calibrated state-space model forward over that period; the retained hardiness was read from the model state at the end of the 10 days. The warm-spell profile was chosen to represent a plausible midwinter thaw for Fargo, and results for 5-day and 15-day thaws are given in **Supplementary Table S5**. Losses are expressed as a percentage of the acclimated hardiness gained above the deacclimated baseline, so that genotypes of differing absolute hardiness are comparable. Across the population the modelled 10-day loss ranged from essentially zero to 43% of acclimated hardiness and was positively correlated with the Deacclimation Risk Index (r = 0.44); the three short-day/low-temperature-responsive genotypes lost the least (9–16%). All curve fitting used non-linear least squares (Levenberg–Marquardt and differential-evolution optimization), and parameter uncertainty was quantified by 1000 non-parametric bootstrap resamples, from which 95% confidence intervals are reported. For the Weibull model in particular, the population-level fit rests on only five NDSU field sampling points per genotype, which is close to the minimum required to identify a three-parameter function; genotype-level shape parameters are therefore reported with their bootstrap confidence intervals and are interpreted qualitatively rather than as precise estimates.

Classifier performance was evaluated by repeated stratified five-fold cross-validation (five folds, ten repeats): within each fold the support-vector machine was trained on four-fifths of the genotypes and its accuracy assessed on the held-out fifth, with folds stratified by hardiness class so that every class was represented in both partitions, and the accuracy was averaged over the 50 resulting fits. No genotype contributed to both the training and the validation partition of any given fold. Bud survival as a function of minimum temperature (**Figure 2C**) was derived directly from the DTA records: for each genotype, sampling time and target temperature, a bud was scored as killed if its low-temperature exotherm occurred at or above that temperature, and the surviving fraction was calculated as the proportion of the 24 buds analyzed whose LTE lay below it; the curves shown are the mean surviving fraction across the 53 genotypes, and no parametric survival model was fitted. Finally, the among-genotype variance of each sampling condition is reported directly, in the units of the trait, as an empirical description of which conditions discriminate most strongly among genotypes. We no longer convert this quantity into a normalized “QTL-readiness score”, since that composite index was not validated against mapping outcome; the underlying variances are shown instead, and the inference drawn from them is limited to the statement that conditions with larger among-genotype variance offer greater power to detect genotypic differences.

## 3 Results

### 3.1 Descriptive statistics of LTE values across sampling times

The mean LTE values differed significantly among the nine sampling times (Tukey’s HSD, p < 0.05), and the sampling times were assigned to four significance groups. The lowest mean LTE values (highest hardiness) were recorded in the two February field samplings, FF-1 and FF-2, at −22.7 °C.

These were followed by the December field samplings DF-1 (−18.3 °C) and DF-2 (−18.6 °C) and the California second sampling C-2 (−17.9 °C). The California first sampling C-1 (−16.5 °C) and the November field sampling NF-1 (−16.6 °C) formed group c. The highest mean LTE values (lowest hardiness) were recorded in the two greenhouse samplings, G-1 (−14.6 °C) and G-2 (−14.7 °C). The widest range between minimum and maximum LTE values occurred under the greenhouse conditions (15.6 °C for G-1 and 15.5 °C for G-2), and the narrowest range occurred in the California first sampling (5.5 °C for C-1). The most negative individual LTE value (−27.1 °C) was recorded in FF-1, and the least negative individual value (−9.0 °C) was recorded in G-1.

### 3.2 Comparison of fitted models

Five mathematical and statistical models were fitted to the cold-hardiness data (**Table 1**). The logistic model of hardening over calendar time and the quadratic response-surface model both explained 99% of the variation (R² = 0.99). The logistic model estimated an asymptotic hardiness of −23.6 °C, a hardening rate of k = 0.30 wk^-^¹, and an inflection point at t_0_ = 7.4 weeks. The response-surface model returned a positive linear chilling coefficient (β_2_ = +11.7) and a negative quadratic chilling coefficient (β_22_ = −8.5). The Weibull model on the accumulated-chilling axis explained 75% of the variation (R² = 0.75), with a scale parameter of λ = 273 chilling degree-days, a shape parameter of κ = 0.93, and a half-hardening point at τ_50_ = 184 chilling degree-days. The mechanistic ODE model explained 57% of the variation (R² = 0.57) and estimated a photoperiod gain of aP = 0.11 and a chilling gain of aC = 1.00. The support-vector machine classifier assigned the genotypes to five hardiness classes using the first two principal components (PC1 = 38.7%, PC2 = 23.8%); the resubstituting (training) accuracy was 83%, but the cross-validated accuracy under repeated stratified five-fold cross-validation was 0.65 ± 0.09, and only the latter is interpreted below. The Weibull parameters are reported with bootstrap 95% confidence intervals in **Supplementary Table S6**. Hierarchical cluster analysis (Ward’s method) of the LTE profiles separated the 53 genotypes into distinct clusters, shown by the dendrogram and heatmap (**Figure 3**).

**Table 1:** Models fitted to the cold-hardiness data.

Model	Equation	Key parameters	R²/Acc.
Logistic	H(t) = H_max_ + (H_min_ − H_max_)/[1 + e^k(t − t0)^]	H_max_ = 23.6 °C; k = 0.30 wk^−1^; t_0_ = 7.4 wk	0.99
Mechanistic ODE	dH/dt = a_P_φ(H_max_−H) + a_C_χ (H_max_−H) – d (H−H_min_)	a_P_ = 0.11; a_C_ = 1.00; d = 0.45	0.57
Response surface	H = β_0_ + β_1_t + β_2_c + β_11_t^2^ + β_22_c^2^ + β_12_tc	β_2_ = +11.7; β_22_ = −8.5	0.99
Weibull (thermal time)	H = H_min_ + (H_max_−H_min_)[1 − e^−(τ/λ)κ^]	λ = 273 CDD; κ = 0.93; τ_50_ = 184 CDD	0.75
SVM classifier (RBF)	f(x) = sign (Σ_i_ α_i_y_i_ K(x_i_,x) + b)	5 hardiness classes; PC1 = 38.7%, PC2 = 23.8%	0.65*

H, hardiness (= −LTE); t, hardening time; c, chilling intensity; τ, accumulated chilling (degree-days, base 5 °C); CDD, chilling degree-days. *Cross-validated accuracy of the support-vector machine (SVM) classifier with a radial basis function (RBF) kernel, obtained by repeated stratified five-fold cross-validation; the resubstitution (training) accuracy was 0.83.

**Figure 3:**
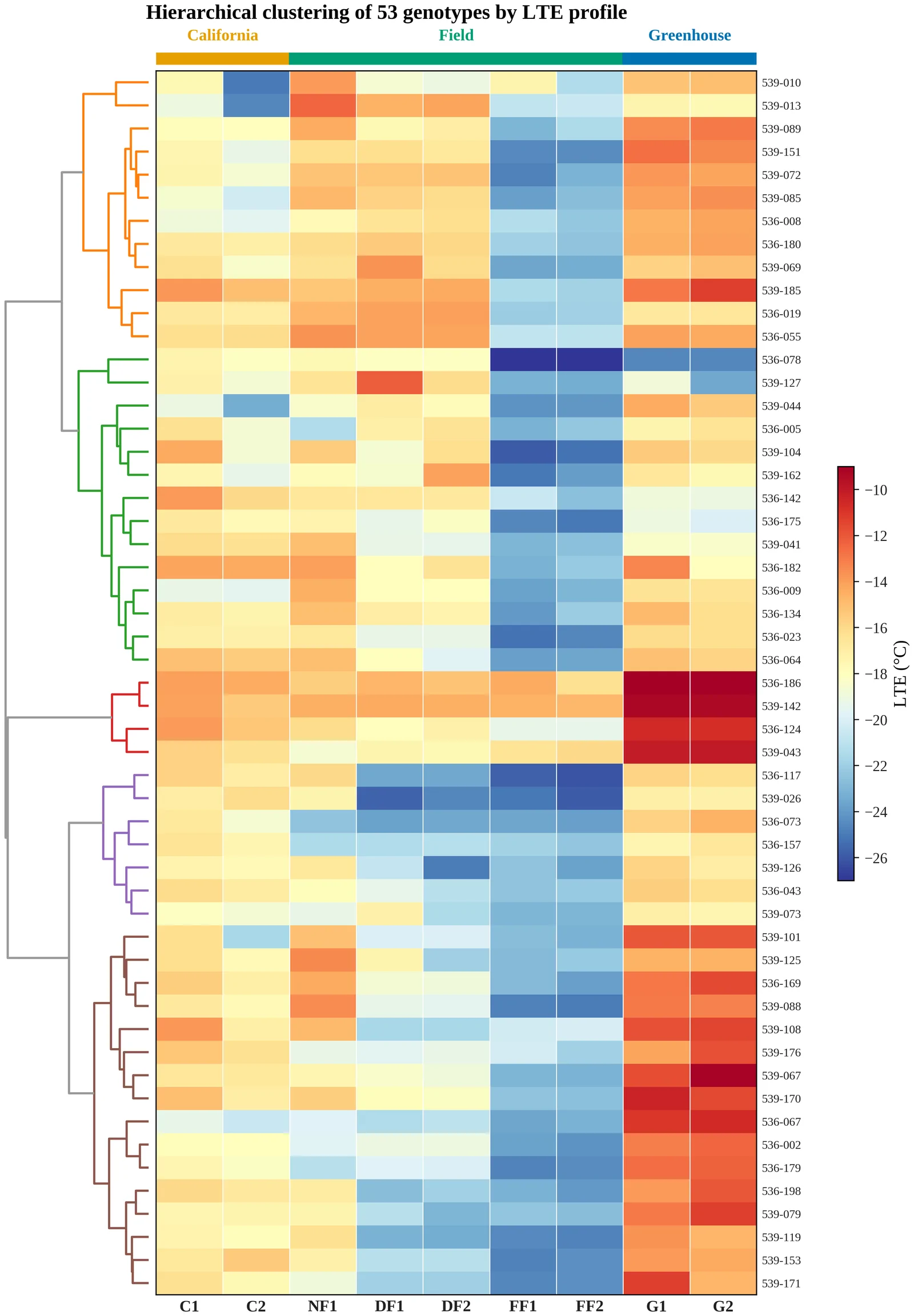
Hierarchical clustering of F_1_ genotypes based on LTE profiles.

### 3.3 Hierarchical clustering of LTE profiles

Across the nine sampling times, the lowest LTE values (highest hardiness) occurred in the February field samplings (FF-1, FF-2), where most genotypes fell between −21 °C and −27 °C, and the highest LTE values (lowest hardiness) occurred in the greenhouse samplings (G-1, G-2). The genotype 536–078 displayed the lowest LTE values across the February field and greenhouse samplings, clustering separately from the rest of the population. The genotypes 536-186, 539-142, 536-124, and 539–043 formed a cluster characterized by consistently high LTE values across the sampling times, particularly under greenhouse conditions. The two December field samplings (DF-1, DF-2) and the two greenhouse samplings (G-1, G-2) each showed concordant patterns within their respective environments. The California field samplings (C-1, C-2) and the November field sampling (NF-1) showed intermediate LTE values across most genotypes.

### 3.4 Logistic modeling of cold acclimation dynamics

The cold acclimation of the field-grown vines was described by a logistic hardening model fitted to the mean low-temperature exotherm (LTE) values, LTE(*t*) = *L*_max_ + (*L*_min_ − *L*_max_)/(1 + *e*^k(t−t0)^). The model fit the NDSU field trajectory with R^2^ = 0.99. The deacclimated baseline (*L*_min_) was −16.0 °C, recorded at the November sampling.

LTE values decreased progressively with hardening time, reaching an asymptotic maximum hardiness (*L*_max_) of −23.6 °C. The hardening rate constant (*k*) was 0.30 wk^−1^, and the inflection point (*t*_0_), at which the acclimation rate was maximal, occurred at 7.4 weeks from the November sampling, corresponding to the late-December to January period. Logistic fits to each of the 53 individual genotypes yielded genotype-specific *L*_max_ values with a population mean of −23.5 °C, and these values ranged from approximately −27 °C to −14 °C across the population. The California vineyard vines recorded mean LTE values of −16.5 °C at the first sampling and −17.9 °C at the second sampling, positioning them in the early-to-intermediate region of the acclimation curve. The NDSU greenhouse vines recorded mean LTE values of −14.6 °C and −14.7 °C at the two sampling times, which fell below the field acclimation curve across the full hardening axis. The maximum LTE difference between the deacclimated baseline and the asymptotic hardiness level was 7.6 °C for the NDSU field environment.

### 3.5 Mechanistic partitioning of photoperiod and chilling contributions

A mechanistic state-space model partitioned the photoperiod- and chilling-driven components of cold acclimation, jointly calibrated to the field and greenhouse environments (**Figure 4**). The photoperiod gain was aP = 0.11, the chilling gain was aC = 1.00, and the deacclimation coefficient was d = 0.45, with a field fit of R² = 0.57. The chilling gain exceeded the photoperiod gain by approximately ninefold. The greenhouse trajectory, driven by the photoperiod term alone, reached an asymptotic hardiness of 16.5 °C (H), whereas the field trajectory, driven by both terms, reached 23.2 °C; the difference attributable to chilling was 6.7 °C. The greenhouse observations fell on the photoperiod-only trajectory, and the California observations occupied the early region of the acclimation curve under mild-winter conditions.

**Figure 4:**
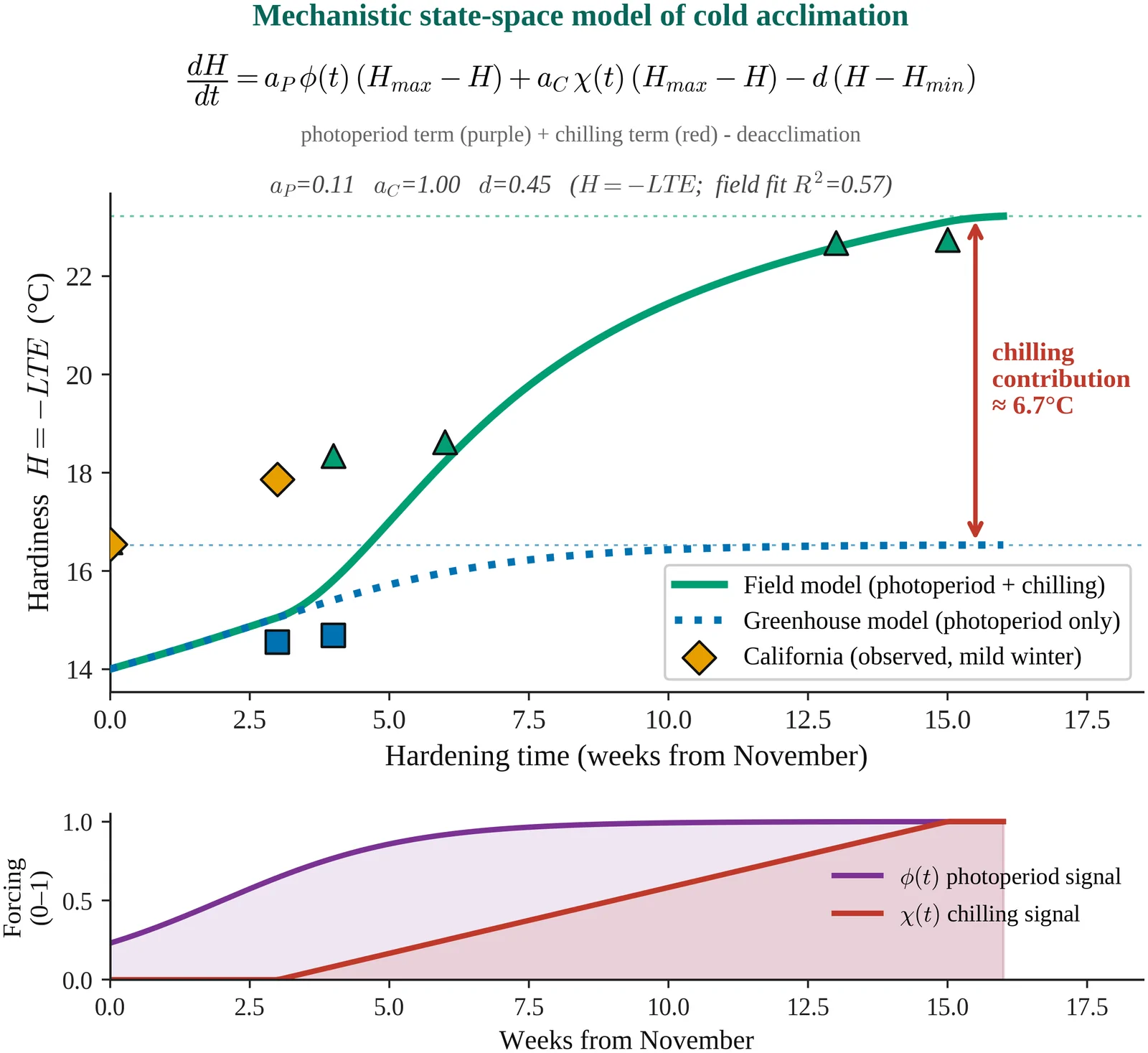
State-space model of photoperiod and chilling contributions.

### 3.6 Response-surface modeling of hardening time and chilling intensity

A second-order response-surface model described bud hardiness as a joint function of hardening time and chilling intensity, explaining 99% of the variation (R² = 0.99). The linear chilling coefficient was large and positive (β2 = +11.7) and the quadratic chilling coefficient was negative (β22 = −8.5). The maximum predicted hardiness, approximately 24 °C, occurred at the longest hardening time combined with full field-level chilling. The lowest-hardiness region corresponded to lowest chilling intensity (greenhouse), where increasing hardening time alone did not raise hardiness to the maximum. The greenhouse conditions occupied the low-hardiness region, the California conditions the intermediate region, and the field conditions extended into the high-hardiness region (**Figure 5**).

**Figure 5:**
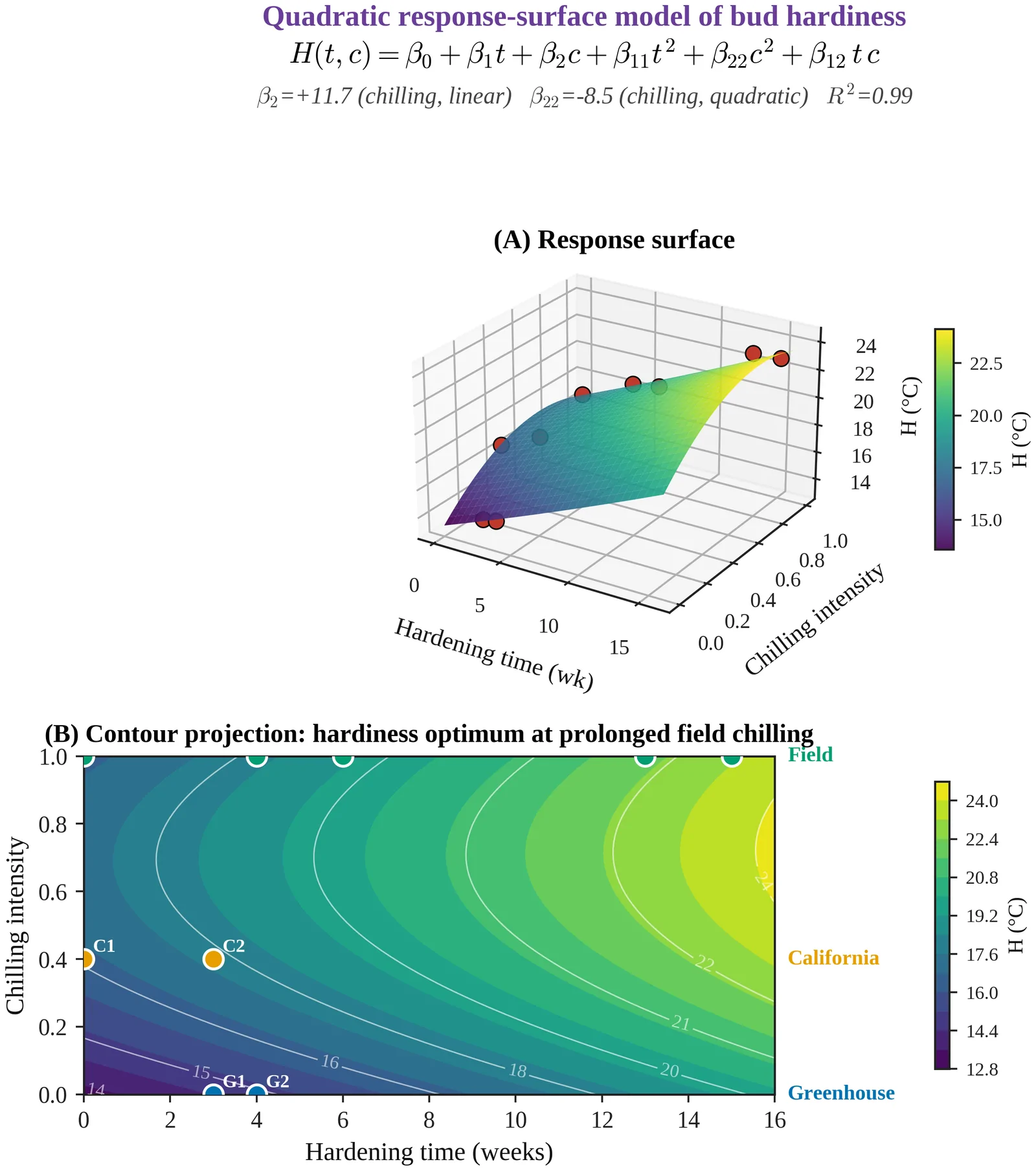
Response-surface model of bud hardiness. **(A)** Three-dimensional quadratic response surface of bud hardiness (H = −LTE, °C) as a joint function of hardening time (weeks) and chilling intensity. **(B)** Contour projection of the same surface, with the California (C-1, C-2), greenhouse (G-1, G-2) and NDSU field samplings overlaid. Maximum hardiness occurs at the longest hardening time combined with full field-level chilling.

### 3.7 Weibull hardening kinetics on a thermal-time axis

Bud cold hardiness was modelled as a function of accumulated chilling using a Weibull cumulative function, with hardiness increasing from a baseline (Hmin = 14 °C) toward a ceiling (Hmax = 24 °C). These values were fitted on the accumulated-chilling axis and therefore differ from the extremes fixed a priori in the state-space model (27 and 13 °C; Section 2.5). At the population level the scale parameter was λ = 273 chilling degree-days and the shape parameter was κ = 0.93 (R² = 0.75). The half-hardening point occurred at τ50 = 184 chilling degree-days. Weibull fits to the 53 individual genotypes yielded shape parameters with a median of κ = 1.25, ranging from below 1 to above 5; genotypes with κ < 1 showed a rapid initial response that decelerated, whereas genotypes with κ > 1 showed a delayed, sigmoidal response. The field-observed values fell along the fitted population curve across the full range of accumulated chilling (**Figure 6**).

**Figure 6:**
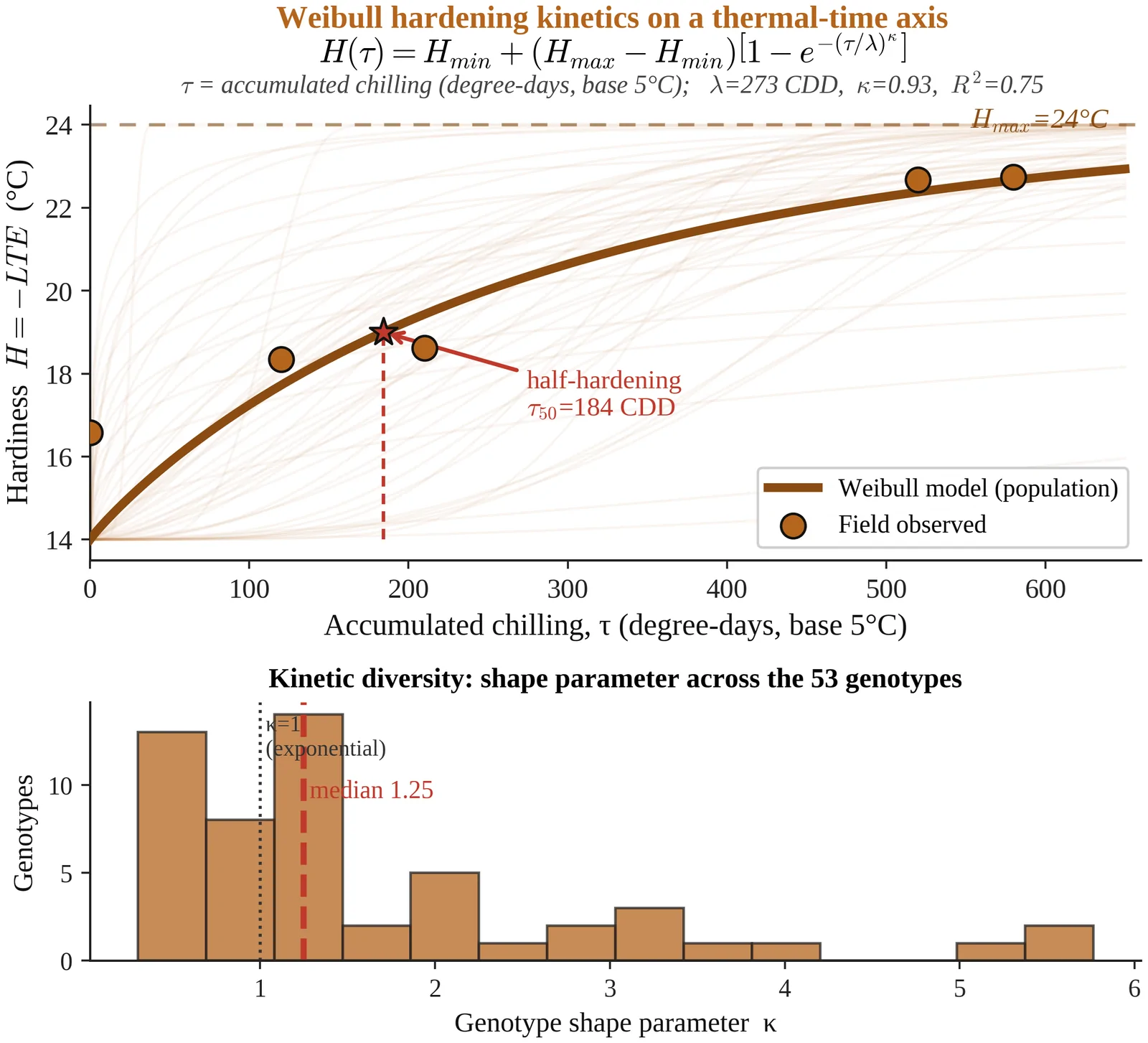
Weibull model of cold-hardening kinetics.

### 3.8 Support-vector classification of cold-hardiness phenotypes

A support-vector machine with a radial basis function kernel classified the genotypes into five cold-hardiness classes from LTE profiles reduced to two principal components (PC1 = 38.7%, PC2 = 23.8%).

The resubstitution accuracy was 83%, but this is an upwardly biased estimate because the classifier was evaluated on the same observations used to fit it; under repeated stratified five-fold cross-validation (five folds, ten repeats) the accuracy was 0.65 ± 0.09. The classifier is accordingly presented as a descriptive partition of the phenotype space rather than as a predictive model, and no claim of generalization to unobserved genotypes is made. The decision boundaries partitioned the phenotype space into five contiguous regions. The very hardy class occupied the negative-PC1 region, containing 536-078, 539-026, and 536-073, whereas the very susceptible class occupied the positive-PC1 region, with 539-142, 536-186, and 539–043 at its periphery. The moderate class formed a transitional band, and the classes were ordered monotonically along the PC1 axis (**Figure 7**).

**Figure 7:**
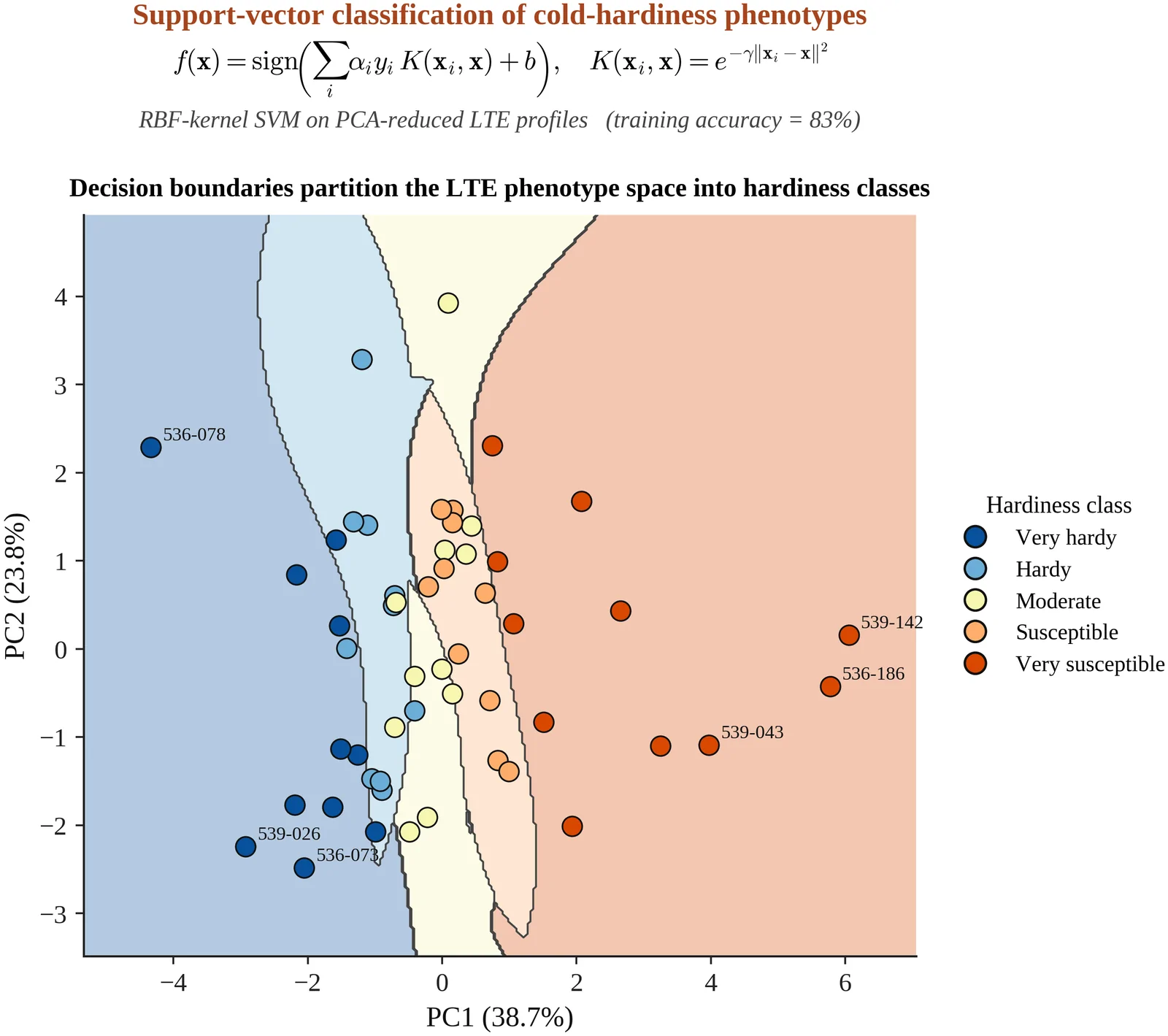
Support-vector classification of cold-hardiness classes.

### 3.9 Relationship between cold hardiness and phenotypic plasticity

Mean cold hardiness and phenotypic plasticity (the LTE range across the three environments) were not significantly correlated (r = 0.15, p = 0.27).

Partitioning at the median of each trait yielded four groups: 15 genotypes were hardy and stable, 11 hardy but plastic, 11 susceptible and stable, and 16 susceptible and plastic. The hardy-and-stable group, combining high hardiness with low environmental variation, included 539-162, 536-009, 536-005, 539-073, 536-175, 539-127, and 539-041. The absence of a significant correlation does not establish that the two traits are independent; it fails to reject the null hypothesis of no linear association, and with 53 genotypes the analysis has limited power to detect a weak relationship. The result is therefore reported as an absence of evidence for a hardiness–plasticity trade-off in this population, and the existence of genotypes in the hardy-and-stable quadrant, rather than the non-significant correlation itself, is what indicates that the two properties can co-occur (**Figure 8**).

**Figure 8:**
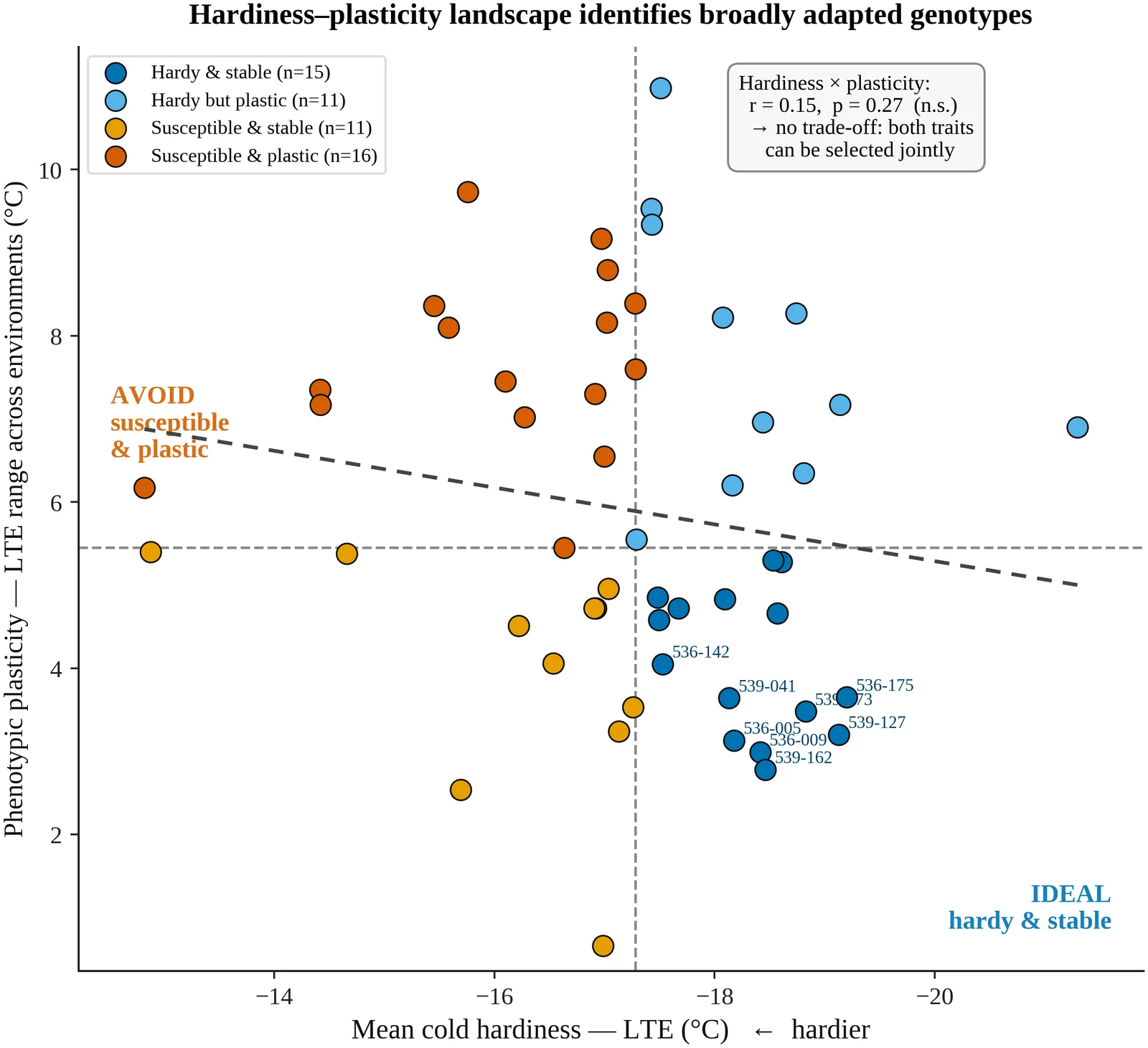
Hardiness–plasticity landscape of the F_1_ population. Mean cold hardiness (LTE averaged across the three growing environments) is plotted against phenotypic plasticity (LTE range across those environments). Genotypes are grouped at the median of each axis into four classes; the dashed line and statistics show the non-significant hardiness–plasticity relationship.

### 3.10 Partitioning of photoperiod- and chilling-driven hardiness among genotypes

A Photoperiod Response Index (PRI) was defined as the proportion of maximum hardiness attained under the greenhouse short-day/moderate-cooling regime, in which temperature did not fall below 4 °C, relative to February field conditions, in which short days were combined with sustained sub-zero chilling. As set out in Section 2.2, the greenhouse regime is not a photoperiod-only treatment, and the index therefore quantifies the hardiness achievable without sub-zero exposure rather than the hardiness attributable to day length in isolation; genotypes with a high index are described below as short-day/low-temperature responsive. The index ranged from 0 to 0.81. Three genotypes were short-day/low-temperature responsive (PRI ≥ 0.65): 536-078 (0.81), 539-127 (0.77), and 536-142 (0.66). Three were intermediate (539-013, 536-175, 539-041), and the remaining 47 were chilling-dependent (PRI < 0.40). The genotype 536–078 recorded the highest hardiness under both greenhouse and field conditions together with the highest PRI. For chilling-dependent genotypes the difference between greenhouse and field hardiness was large, whereas for photoperiod-responsive genotypes it was small (**Figure 9**).

**Figure 9:**
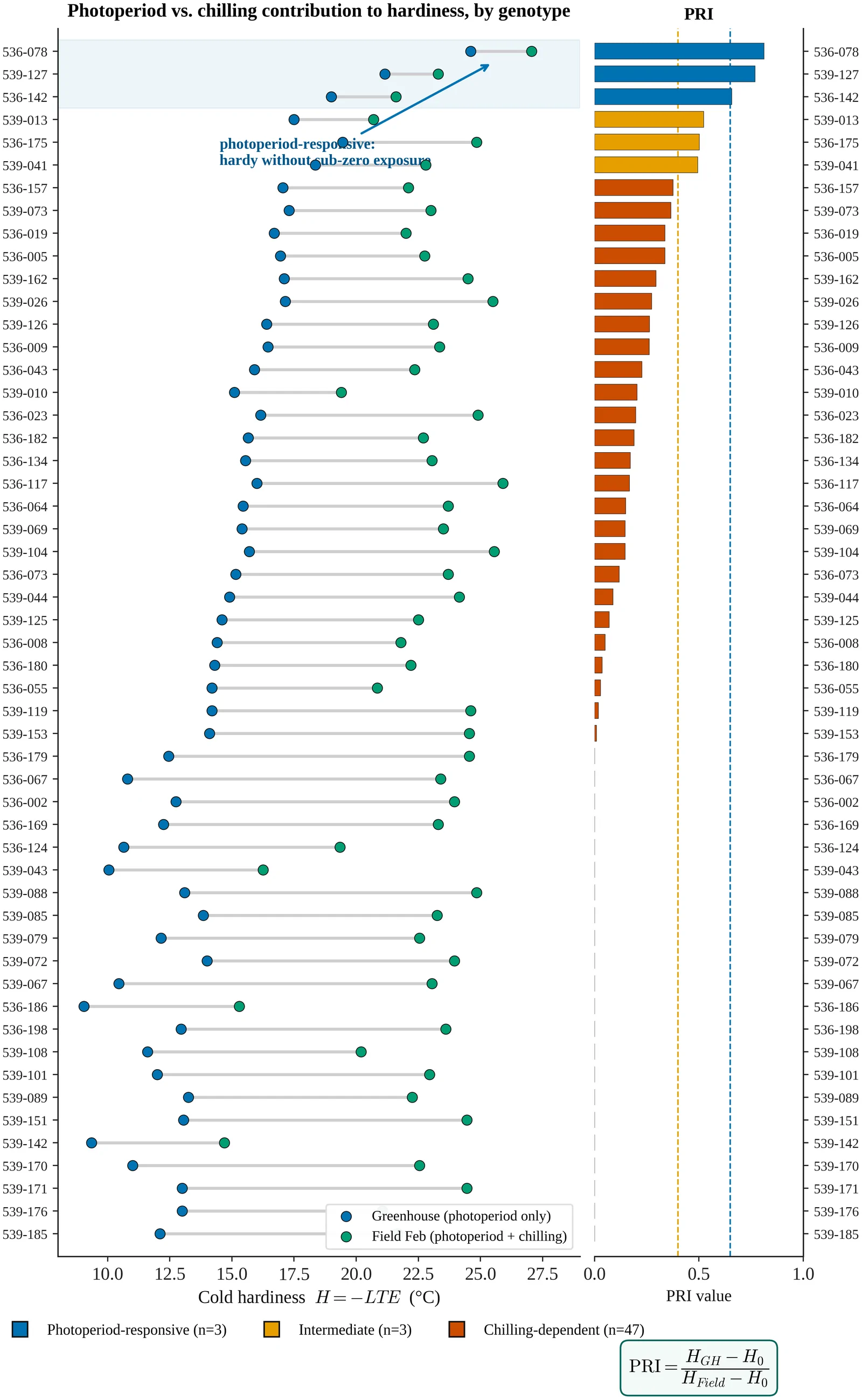
Photoperiod Response Index across genotypes. For each genotype, the NDSU greenhouse hardiness (short days with moderate cooling, no sub-zero exposure) and the NDSU field February hardiness (short days with sustained sub-zero chilling) are shown; PRI is the proportion of maximum hardiness attained without sub-zero exposure. H_GH_, greenhouse hardiness; H_Field_, field hardiness; H_0_, baseline. NDSU, North Dakota State University.

### 3.11 Variance partitioning, segregation, survival, and correlation structure

The variance in bud cold hardiness was partitioned into environmental (40%), residual (48%), genotypic (7%), and genotype × environment (5%) components. The single-observation broad-sense heritability was accordingly low (*H*², *i* = 0.07), whereas the entry-mean heritability of a genotype means taken over three environments and four replicates was moderate (*H*², *c* = 0.49). The two values are internally consistent given *e* = 3 and *r* = 4 (Section 2.5): the entry-mean statistic describes the reliability of a genotype mean averaged over environments and replicates, and is therefore not comparable with the 7% genotypic variance component.

Nine genotypes were hardier than the tender parent (*V. vinifera*) but none exceeded the hardy parent (*V. riparia*), so all progeny values fell between the parental extremes. This represents segregation toward the hardier parent rather than transgressive segregation in the strict sense. The temperature at which mean bud survival across genotypes fell to 50% was approximately −20 °C for the field, −17 °C for California, and −15 °C for the greenhouse, with the field maintaining the highest surviving fraction across the temperature range. The correlation network showed four within-group clusters (FF-1/FF-2, G-1/G-2, DF-1/DF-2, C-1/C-2), while the November field sampling (NF-1) showed no correlation above the threshold with any other sampling (**Figure 2D**). This is not inconsistent with the assignment of NF-1 and C-1 to the same Tukey group c in **Table 2**: the compact-letter display groups sampling times whose population mean LTE values do not differ significantly, whereas the correlation network describes whether the rank order of genotypes is preserved between two samplings. NF-1 and C-1 have statistically indistinguishable means (−16.6 and −16.5 °C) but rank the genotypes differently (r = 0.18), so that the two analyses answer different questions and can legitimately disagree.

**Table 2:** Descriptive statistics of LTE values across nine sampling times.

Sampling time	Mean ± SEM (°C)	Minimum (°C)	Maximum (°C)	Range (°C)	Group
C-1	-16.5 ± 0.20	-19.3	-13.8	5.5	*c*
C-2	-17.9 ± 0.30	-25.0	-14.3	10.7	*b*
NF-1	-16.6 ± 0.31	-22.5	-12.4	10.1	*c*
DF-1	-18.3 ± 0.39	-25.6	-12.2	13.4	*b*
DF-2	-18.6 ± 0.40	-24.9	-14.0	10.9	*b*
FF-1	-22.7 ± 0.35	-27.1	-14.3	12.8	*a*
FF-2	-22.7 ± 0.31	-27.0	-14.8	12.2	*a*
G-1	-14.6 ± 0.39	-24.6	-9.0	15.6	*d*
G-2	-14.7 ± 0.45	-24.6	-9.1	15.5	*d*

Values are means of 53 genotypes ± standard error of the mean (SEM). Means in the “Group” column followed by the same letter are not significantly different (Tukey’s HSD, p < 0.05). C, California (Parlier-grown, cold-stored); NF, November field; DF, December field; FF, February field; G, greenhouse sampling; NDSU, North Dakota State University.

### 3.12 Deacclimation risk and phenotyping power

A Deacclimation Risk Index (DRI), the fraction of peak hardiness dependent on sustained chilling and hence predicted to be lost during the simulated 10-day, 10 °C midwinter thaw defined in Section 2.5, ranged from 0.19 to 1.00. The genotypes 536-078 (0.19), 539-127 (0.23), and 536-142 (0.34) had the lowest values and retained most of their hardiness after the modelled warm spell, plotting close to the 1:1 line; a group including 536-002, 536-124, 536-169, 536-179, and 536–067 had a DRI of 1.00 and showed the largest modelled loss of hardiness. The among-genotype variance in LTE differed substantially among sampling conditions, being highest for the greenhouse second sampling (G-2, σ² = 10.6 °C²) and the December field samplings (DF-2, σ² = 8.7 °C²; DF-1, σ² = 8.2 °C²), and lowest for the California first sampling (C-1, σ^2^ = 2.2 °C^2^, which is additionally affected by the storage confound described in Section 2.3); in **Figure 10B** these variances are displayed scaled to the largest value, with the absolute variance printed beside each bar. Conditions with larger among-genotype variance provide greater statistical power to detect genotypic differences and are therefore the more informative phenotyping windows; we make no further claim about mapping outcomes, since no mapping was performed here.

**Figure 10:**
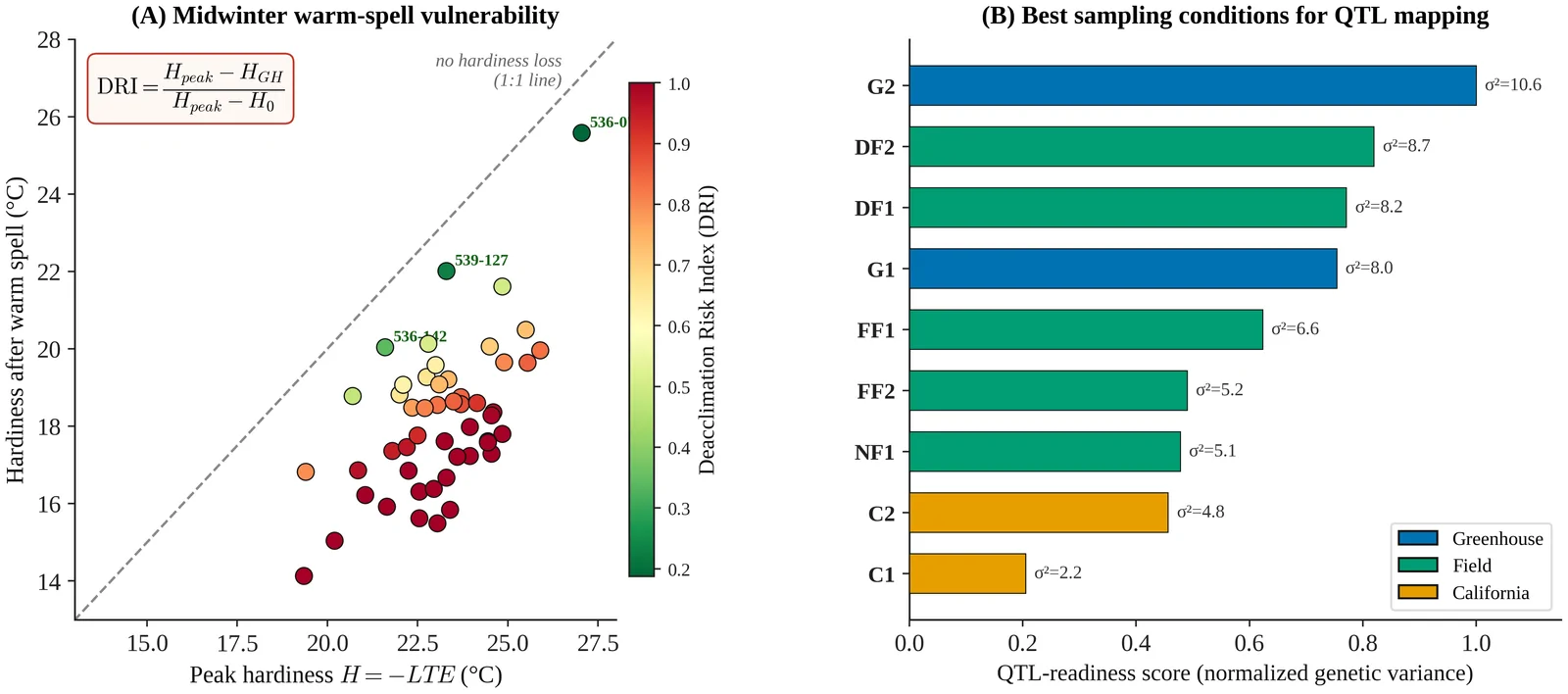
Midwinter warm-spell vulnerability and phenotyping power. **(A)** Hardiness after a simulated 10-day, 10 °C midwinter warm spell plotted against peak hardiness (H = −LTE, °C) for each genotype; points are coloured by the Deacclimation Risk Index (DRI) and the dashed line is the 1:1 line of no hardiness loss. **(B)** Among-genotype variance in LTE for each of the nine sampling conditions, coloured by environment (greenhouse, field, California). Bars are scaled to the largest value (G-2) and the absolute variance (σ^2^, °C^2^) is printed beside each bar; the axis label "QTL-readiness score" refers only to this scaling of the among-genotype variance and implies no claim about mapping outcome, since no QTL mapping was performed. Conditions with larger among-genotype variance provide greater power to detect genotypic differences.

## 4 Discussion

### 4.1 Genetic variation and the genetic basis of cold hardiness

The present results revealed substantial diversity in cold hardiness within the V. riparia × V. vinifera F_1_ population and provided a quantitative first step toward dissecting the genetic basis of this trait (**Table 2**; **Figure 3**). Mean genotype LTE values spanned −13.4 to −21.4 °C, and the population separated into five distinct hardiness classes ranging from cold-sensitive to cold-hardy (**Figure 7**). The “very cold-hardy” class, comprising genotypes such as 536-078, 539-026, and 536–073 with LTE values as low as −21.4 °C, represents a promising resource for introgression superior hardiness into elite cultivars. Critically, our analysis advanced beyond this descriptive ranking by partitioning the phenotypic variance into its genetic and environmental components (**Figure 2A**). Genotype accounted for only 7% of the total variance and the genotype × environment interaction for a further 5%, whereas environment and residual variation dominated. The implication of these components must be stated carefully. A single LTE observation on a single genotype in a single environment carries little genetic information (*H*², *i* = 0.07), and selection on such an observation would be very inefficient. Averaging over three environments and four replicates, however, raises the reliability of the genotype mean substantially (*H*², *c* = 0.49), because the environmental and residual contributions to the mean are divided by the number of observations. The correct reading is therefore not that a large genetic component underlies the trait, but that the modest genetic component can be estimated with moderate reliability provided that genotypes are phenotyped across several environments and replicates. This distinction matters for breeding practice, since it implies that multi-environment phenotyping is a precondition for effective selection in this population rather than an optional refinement. This reconciles two observations that are often treated separately in the grapevine literature: that cold hardiness is highly environmentally labile (Londo and Kovaleski, 2017; Kaya and Kose, 2020; Kose et al., 2026), and yet that hardy selections can be reliably recovered from interspecific crosses (Hemstad and Luby, 2002). The segregation we observed (**Figure 2B**), in which nine genotypes were hardier than the tender parent while none exceeded the hardy parent, is the expected signature of such a heritable, polygenic trait, reflecting the reassortment of complementary alleles from the two parental species (Falconer and Mackay, 1996).

### 4.2 Environmental control of cold acclimation across sites and sampling times

The variation in LTE across growing environments and sampling times underscored the intricate interplay between genotype and environment in governing acclimation and deacclimation (**Figure 11**). In the NDSU field, hardiness increased progressively as winter advanced: the November and December samplings were comparatively unhardened (−16.6 and −18.3 to −18.6 °C, respectively), whereas both February samplings reached a maximum of −22.7 °C. This increase of approximately 6 °C between autumn and midwinter confirms the central role of prolonged exposure to low temperature in driving acclimation (Keller and Mills, 2007; Xiao et al., 2008; De Rosa et al., 2021; Xin and Browse, 2000). The California samples, by contrast, attained only intermediate hardiness (−16.5 and −17.9 °C), which is consistent with reports that warm temperatures and prolonged growing seasons delay or impede acclimation (Fennell and Hoover, 1991), but which must be interpreted with the storage confound of Section 2.3 in mind: these buds were analyzed after one and four weeks at 4 °C respectively, so that part of the difference between C-1 and C-2, and part of the difference between the California and Fargo values, reflects post-collection storage rather than acclimation in the Parlier vineyard. The wider LTE range at the second California sampling (10.7 °C) than at the first (5.5 °C) further indicates that the specific temperature conditions experienced during the acclimation window strongly shape the expressed phenotype (Londo and Kovaleski, 2017; Xiao et al., 2008). Notably, the greenhouse samplings produced the lowest mean hardiness (−14.6 and −14.7 °C) yet the widest within-environment range (15.6 °C), demonstrating that a controlled short-day and moderate-cooling regime, in the absence of sub-zero exposure, does not reproduce the field acclimation response but does expose considerable genotypic variation (Keller and Mills, 2007; De Rosa et al., 2021). The correlation network (**Figure 2D**) clarified this temporal structure: samplings within an environment were tightly correlated (February–February, December–December, greenhouse–greenhouse), whereas the November sampling was uncorrelated with all others, indicating that early-autumn hardiness is a poor predictor of midwinter hardiness and that genotypes are not yet differentiated at that stage.

**Figure 11:**
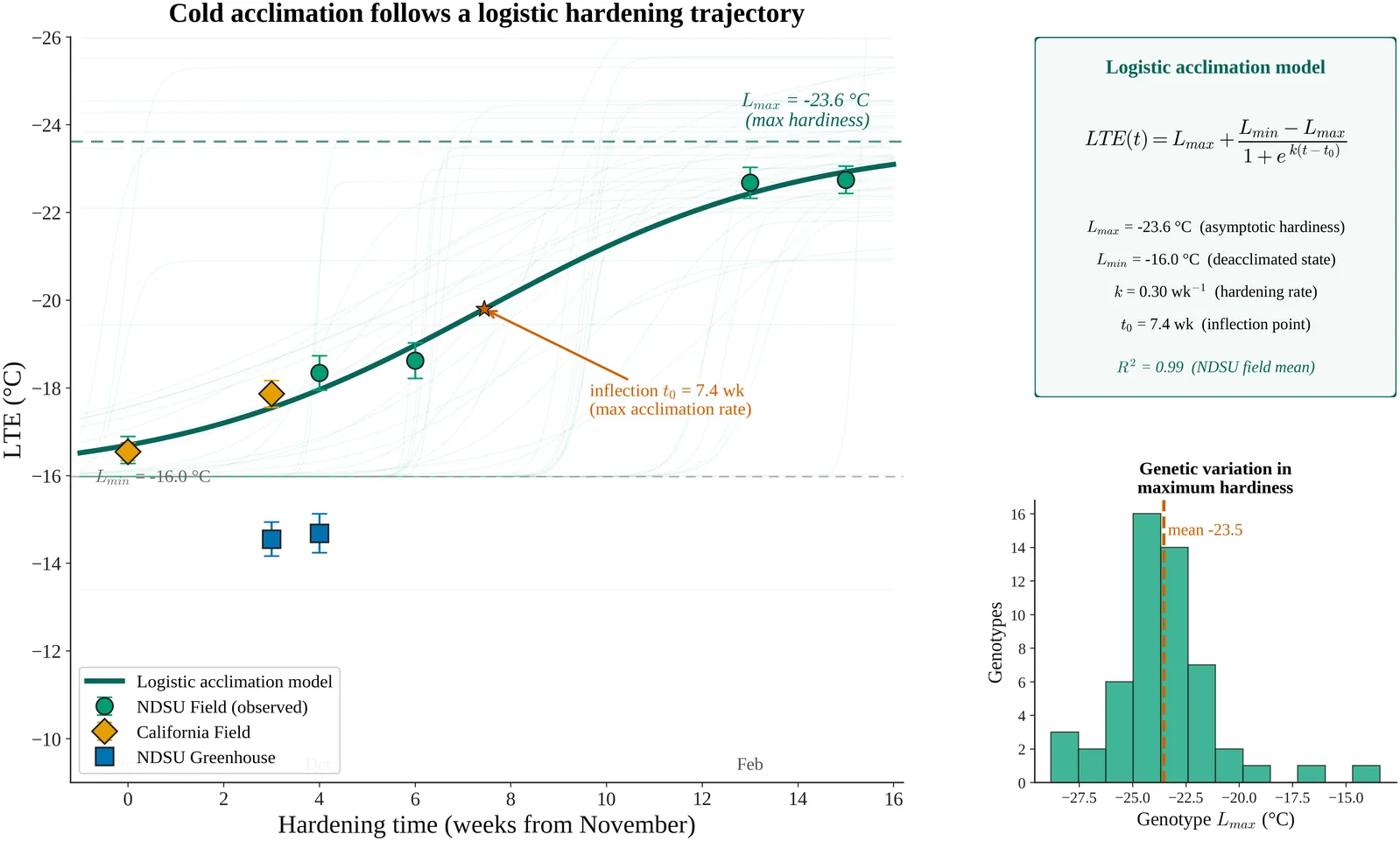
Logistic model of cold acclimation. Fitted to the mean LTE of NDSU field-grown vines, with California vineyard and greenhouse samples overlaid. The curve gives the asymptotic hardiness (L_max_), deacclimated baseline (L_min_), hardening rate (k) and inflection point (t_0_); faint lines show individual genotype fits and the histogram shows the distribution of genotype L_max_ values. NDSU, North Dakota State University.

### 4.3 Partitioning photoperiod- and chilling-driven hardiness

A central contribution of this study was the quantification of the hardiness conferred by sustained sub-zero chilling over and above a short-day, moderate-cooling regime, achieved by contrasting the greenhouse (short days, no sub-zero exposure) with the field (short days plus sustained sub-zero chilling) and formalized in a mechanistic state-space model (**Figure 4**). We stress that this is not a clean separation of photoperiod from temperature: both cues varied between the two environments, so that the quantity estimated is the incremental effect of sub-zero chilling within a design in which the two environments also differed in photoperiod trajectory and in absolute temperature above zero. The model attributed a far larger gain to chilling (a_C = 1.00) than to photoperiod (a_P = 0.11), and the difference between the greenhouse asymptote (−16.5 °C) and the full field asymptote (−23.2 °C) placed the contribution of sustained sub-zero chilling at approximately 6.7 °C. Because a_C is estimated from a between-environment contrast in which photoperiod is not held constant, this figure should be read as an upper bound on the chilling effect within this design. This quantitative partitioning provides a mechanistic basis for the long-standing, largely qualitative distinction in the literature between photoperiod- and temperature-driven dormancy induction (Schnabel and Wample, 1987; Fennell and Hoover, 1991; Wake and Fennell, 2000). Crucially, genotypes differed in their reliance on these cues. The Photoperiod Response Index (**Figure 9**) identified three genotypes, 536-078, 539-127, and 536-142, that attained 66–81% of their maximum hardiness under short days combined with moderate cooling and without any sub-zero exposure, whereas the remaining genotypes required sustained chilling. This division is consistent with, rather than a direct corroboration of, the classic work of Schnabel and Wample (1987) and Wake and Fennell (2000): Schnabel and Wample reported that short days act synergistically with declining temperature and explicitly attributed no acclimation to short days alone before the onset of low night temperatures, and Wake and Fennell examined growth cessation and dormancy rather than bud cold acclimation. Our data extend that work by showing that genotypes within a single segregating family differ markedly in how much hardiness they can attain without sub-zero exposure, but they do not demonstrate acclimation in response to day length in isolation, which the present design cannot test. The physiological processes underlying these responses, accumulation of cryoprotective solutes and LEA-like proteins, membrane remodeling, and induction of cold-responsive genes, are themselves modulated by both photoperiod and temperature (Salzman et al., 1996; Sanghera et al., 2011; Sanghera and Sharma, 2011; Xiao et al., 2008; Londo and Kovaleski, 2017; Rende et al., 2018), and the genotype-specific balance between them is consistent with the combined photoperiod-and-temperature control of acclimation reported by Kalcsits et al. (2009). The existence of genotypes that harden substantially without sub-zero exposure is of particular applied importance, as such material may acclimate more reliably in regions where winters are too mild or too variable to provide a dependable chilling signal. Confirming that day length as such, rather than the accompanying moderate cooling, drives this response will require the orthogonal photoperiod × temperature design outlined in Section 4.6.

### 4.4 Quantitative modeling of the acclimation trajectory

To move from description toward prediction, we characterized the acclimation trajectory with complementary mathematical models (**Table 1**; **Figures 3**, **4**, **5**). The logistic model described the field time course almost perfectly (R² = 0.99), estimating an asymptotic hardiness of −23.6 °C and an inflection point at 7.4 weeks, corresponding to the late-December to January period of maximal hardening rate. This formalizes, as a single interpretable parameter, the well-documented observation that grapevine hardiness develops sigmoidally rather than linearly through winter (Mills et al., 2006; Kalberer et al., 2006). The response-surface model (R² = 0.99) showed that hardiness rose steeply with chilling intensity (β_2_ = +11.7) but with diminishing returns (β_22_ = −8.5), so that maximum hardiness required both prolonged hardening time and full field-level chilling, a quantitative restatement of the principle that maximum hardiness is environment-dependent and not consistently attained across winters (Londo and Kovaleski, 2017). Recasting the field response on an accumulated-chilling axis, the Weibull model (R² = 0.75) placed the half-hardening point at 184 chilling degree-days and indicated genotypic diversity in hardening kinetics (median shape parameter κ = 1.25), with some genotypes apparently responding rapidly and others following a delayed, sigmoidal course. This last inference is the least secure result of the study: each genotype-level Weibull fit rests on five field sampling points, and the bootstrap confidence intervals on κ (**Supplementary Table S6**) are correspondingly wide, with a mean width of 1.05 units and an interval that includes κ = 1 for 24 of the 53 genotypes, so that the distribution of κ should be read as evidence that genotypes differ in kinetics rather than as a set of reliable individual estimates. Denser sampling through the acclimation window would be required to estimate genotype-specific kinetics with confidence. Such thermal-time framing is consistent with the view that acclimation is governed by accumulated cold exposure rather than calendar date (Kalberer et al., 2006; Xiao et al., 2008). Together these models converge on a single conclusion from different mathematical perspectives: hardiness is a saturating function of accumulated chilling, its rate and ceiling vary among genotypes, and the greenhouse photoperiod-only response falls consistently short of the field ceiling, reinforcing the partitioning described above.

### 4.5 Implications for breeding and viticulture under a changing climate

The combined analyses carry direct implications for cultivar development and deployment (Roca et al., 2019). These findings align with work on the regional evaluation of cold-climate cultivars (Atucha et al., 2018; Schrader et al., 2019) and on the biochemical basis of cold acclimation in grapevine buds (Rende et al., 2018; Kaya, 2020). First, the hardiness–plasticity landscape (**Figure 8**) showed that mean hardiness and phenotypic plasticity were uncorrelated (r = 0.15, p = 0.27), providing no evidence that high hardiness and environmental stability are mutually constrained, although a non-significant correlation cannot by itself establish independence; genotypes such as 539-162, 536-009 and 539-073 combined deep hardiness with low cross-environment variation and represent broadly adapted candidates. This is an encouraging result for breeding, because it implies no inherent trade-off between being hardy and being reliable across sites, a distinction rarely quantified in grapevine but well recognized as a goal of genotype-by-environment analysis (Falconer and Mackay, 1996; Wakchaure et al., 2016). Second, the deacclimation-risk analysis (**Figure 10A**) indicated that, under the 10-day, 10 °C thaw simulated in Section 2.5, the most chilling-dependent genotypes are predicted to lose the largest fraction of their midwinter hardiness, whereas the short-day/low-temperature-responsive genotypes (536-078, 539-127, 536-142) are predicted to retain most of theirs. This is a model projection, not an observation: no warm spell was imposed experimentally, and the projection inherits all the assumptions of the state-space model, including the definition of χ(t) and the assumed thaw profile. It is offered as a hypothesis about relative deacclimation risk that merits direct experimental testing, and we draw no conclusion here about performance under climate change. Third, both the descriptive data and the among-genotype variance analysis (**Figure 10B**, in which the bars show the among-genotype variance scaled to its maximum) identified the December field sampling and the greenhouse as the conditions with the greatest among-genotype variance, and therefore as the most informative stages for phenotyping this population. Collectively, these findings support a selection strategy that prioritizes not merely the deepest midwinter hardiness but the combination of high hardiness, low cross-environment variability, and the capacity to acclimate without sustained sub-zero exposure, subject to the experimental confirmation set out below.

### 4.6 Limitations and directions for further work

Four limitations bound the inferences drawn above. First, and most importantly, the three environments differ simultaneously in photoperiod and in temperature, so that the design is not orthogonal with respect to the two cues of interest. The quantity we estimate is the additional hardiness conferred by sustained sub-zero chilling relative to a short-day, moderate-cooling regime; it is not the effect of temperature at constant photoperiod, nor the effect of photoperiod at constant temperature. Resolving these requires a factorial experiment in controlled environments, in which vines of the same genotypes receive a common photoperiod under contrasting temperature regimes and a common temperature under contrasting photoperiods, or a field experiment in which supplemental lighting extends the photoperiod of a subset of vines at a common site. We regard such an experiment as the necessary next step and are planning it for the material described here. Second, the California samples were analyzed after one and four weeks of storage at 4 °C, and therefore index the hardiness of Parlier-grown, cold-stored buds rather than the hardiness of vines in a mild-winter vineyard; the California environment contributes a chilling gradient to the analysis but should not be read as an unbiased mild-winter field treatment. Third, the study covers a single dormant season (2023–2024). Because maximum hardiness is not attained consistently across winters, the environmental variance component, the genotype × environment interaction, and the entry-mean heritability are all specific to the conditions of that season, and the entry-mean heritability in particular would be expected to change with the number and diversity of seasons sampled. Repeating the phenotyping over two or three further dormant seasons would allow year to be included as a separate random term, would test whether the ranking of genotypes and the identity of the short-day/low-temperature-responsive genotypes are stable across winters, and would establish whether the December phenotyping window is consistently the most discriminating one. We expect additional seasons to increase confidence in the genotype rankings rather than to alter them, but this remains to be demonstrated. Fourth, replication differs among environments, being two vines per genotype in the NDSU field, three in the greenhouse and one at Parlier; the California variance components are correspondingly the least well estimated.

A further question raised by these data concerns the genetic architecture of the parental species. The *V. riparia* seed parent is highly cold hardy, as expected of a species distributed across North Dakota, Minnesota and the Canadian prairies, yet the F_1_ population segregates widely for hardiness and includes genotypes considerably more tender than either parent would suggest, with nine genotypes exceeding the tender parent and none exceeding the hardy parent (Minnesota Grape Growers Association, 2016). Several explanations are compatible with the data. Cold hardiness in *Vitis* is polygenic, so a hardy *V. riparia* individual is expected to be heterozygous at many contributing loci and to transmit a sample of both increasing and decreasing alleles; alleles that reduce hardiness need not be deleterious in *V. riparia* if their effect is conditional on genetic background or on the accompanying phenology, since in the wild the species survives through a coordinated syndrome of early growth cessation, deep dormancy and acclimation, and an allele that is neutral or beneficial within that syndrome may appear as a hardiness-reducing allele when placed in a hybrid background with *V. vinifera* phenology. In addition, the F_1_ genotypes inherit half their genome from a species with no history of selection for winter survival, so that hybrid phenotypes reflect the disruption of the *V. riparia* syndrome as much as the segregation of individual hardiness alleles. We therefore do not interpret the tender F_1_ genotypes as evidence that strongly cold-susceptible alleles segregate at high frequency in wild *V. riparia* populations; distinguishing these possibilities would require phenotyping a panel of wild *V. riparia* accessions and their progeny, which is beyond the scope of the present study but is a natural extension of it.

## 5 Conclusion

This study evaluated cold hardiness in a *Vitis riparia* × *V. vinifera* F_1_ population across three contrasting environments and, for the first time in this cross, quantified the genetic and environmental architecture of the trait. Hierarchical clustering resolved five classes of freezing tolerance, from very hardy to very cold susceptible; the entry-mean heritability was moderate (H²c = 0.49) but the single-observation heritability low (H²i = 0.07), indicating that a selectable genetic component exists but can be resolved only through multi-environment, replicated phenotyping. Field vines reached a midwinter maximum of −22.7 °C, whereas greenhouse vines were most susceptible (−14.6 °C), and by contrasting the two environments we estimated that sustained sub-zero chilling contributed approximately 6.7 °C of additional hardiness, an upper bound because the environments also differed in photoperiod. Most genotypes required sustained chilling, but three (536-078, 539-127, 536-142) acquired most of their hardiness without sub-zero exposure and were predicted to be the most resilient to a modelled warm spell, while others such as 536-078, 539–026 and 536–073 combined deep hardiness with cross-environment stability, making them valuable parents for cold-hardy breeding. The December field and greenhouse samplings exposed the widest among-genotype variation and are therefore the most informative phenotyping conditions. Further work should repeat this phenotyping over additional dormant seasons to establish the stability of the genotype rankings and heritability estimates, impose an orthogonal photoperiod × temperature design to test whether the responsive genotypes react to day length as such, and use the December field and greenhouse phenotypes to map QTL for bud cold hardiness in this population. No QTL mapping was undertaken here, and these statements identify the most informative phenotyping conditions for such an effort rather than reporting any mapping result.

## Data Availability

The original contributions presented in the study are included in the article/**Supplementary Material**, further inquiries can be directed to the corresponding author/s.
