# Supplementary material for: Partitioning photoperiod- and chilling-driven cold hardiness in a Vitis riparia × V. vinifera F1 population
Source: Front Plant Sci. 2026 Sep 30;17:1916819. doi: 10.3389/fpls.2026.1916819 (PMC13662123; doi:10.3389/fpls.2026.1916819)
Supplement: Supplementary file 1 [file Table1.docx]

**Table S1.** Calendar dates of the nine sampling times of the 2023–2024 dormant season.

| **Sampling code** | **Environment** | **Collection date** | **Storage or treatment before DTA** | **DTA date** |
| --- | --- | --- | --- | --- |
| NF-1 | NDSU field (Fargo) | 3 Nov 2023 | None; analyzed within 24 h of collection | 3 Nov 2023 |
| DF-1 | NDSU field (Fargo) | 8 Dec 2023 | None; analyzed within 24 h of collection | 8 Dec 2023 |
| DF-2 | NDSU field (Fargo) | 22 Dec 2023 | None; analyzed within 24 h of collection | 22 Dec 2023 |
| FF-1 | NDSU field (Fargo) | 1 Feb 2024 | None; analyzed within 24 h of collection | 1 Feb 2024 |
| FF-2 | NDSU field (Fargo) | 15 Feb 2024 | None; analyzed within 24 h of collection | 15 Feb 2024 |
| C-1 | California vineyard (Parlier) | 7 Jan 2024 | Shipped to NDSU; held 1 week at 4 °C | 14 Jan 2024 |
| C-2 | California vineyard (Parlier) | 7 Jan 2024 | Shipped to NDSU; held 1 week at 4 °C and a further 3 weeks (4 weeks in total) | 4 Feb 2024 |
| G-1 | NDSU greenhouse (Fargo) | 20 Feb 2024 | 8-week regime, then 3 weeks at 4 °C | 20 Feb 2024 |
| G-2 | NDSU greenhouse (Fargo) | 27 Feb 2024 | 8-week regime, then 4 weeks at 4 °C | 27 Feb 2024 |

DTA, differential thermal analysis. C, California vineyard; NF, November field; DF, December field; FF, February field; G, greenhouse. Field samples were analyzed within 24 h of collection, so the DTA date corresponds to the collection date. The December and February samplings were each made on two occasions separated by two weeks, corresponding to the first and third weeks of those months. The California samples were collected on a single date and analyzed after two different periods of storage at 4 °C, as described in Section 2.3; the difference between C-1 and C-2 therefore reflects storage duration in addition to any difference in field acclimation. The greenhouse regime was initiated on a phenological criterion (once vines had reached 12 nodes) rather than on a fixed calendar date, and began on 5 December 2023; G-1 and G-2 follow the start of that regime by 11 and 12 weeks respectively. The week-by-week schedule is given in Supplementary Table S3.

**Table S2.** Daily air temperature, solar radiation and day length at the two outdoor sites, 1 September 2023 to 29 February 2024.

| **Site** | **Latitude (°N)** | **Date** | **Tmax (°C)** | **Tmin (°C)** | **Tmean (°C)** | **Solar radiation (MJ m⁻² d⁻¹)** | **Day length (h)** | **CDD (base 5 °C)** |
| --- | --- | --- | --- | --- | --- | --- | --- | --- |
| Fargo, ND | 46.89 | 1 Sep 2023 | 32.2 | 17.2 | 24.4 | 19.30 | 13.40 | 0.0 |
| Fargo, ND | 46.89 | 2 Sep 2023 | 33.3 | 18.9 | 26.1 | 21.19 | 13.35 | 0.0 |
| Fargo, ND | 46.89 | 3 Sep 2023 | 33.9 | 19.4 | 26.7 | 21.31 | 13.30 | 0.0 |
| Fargo, ND | 46.89 | 4 Sep 2023 | 34.4 | 21.7 | 28.3 | 14.49 | 13.24 | 0.0 |
| Fargo, ND | 46.89 | 5 Sep 2023 | 26.7 | 14.4 | 20.6 | 2.47 | 13.19 | 0.0 |
| Fargo, ND | 46.89 | 6 Sep 2023 | 20.6 | 10.0 | 15.0 | 20.72 | 13.13 | 0.0 |
| Fargo, ND | 46.89 | 7 Sep 2023 | 23.3 | 9.4 | 16.1 | 19.97 | 13.08 | 0.0 |
| Fargo, ND | 46.89 | 8 Sep 2023 | 25.0 | 12.2 | 18.9 | 19.05 | 13.03 | 0.0 |
| Fargo, ND | 46.89 | 9 Sep 2023 | 24.4 | 9.4 | 16.7 | 14.95 | 12.97 | 0.0 |
| Fargo, ND | 46.89 | 10 Sep 2023 | 23.9 | 6.1 | 15.0 | 19.72 | 12.92 | 0.0 |
| Fargo, ND | 46.89 | 11 Sep 2023 | 20.0 | 7.8 | 13.9 | 10.84 | 12.86 | 0.0 |
| Fargo, ND | 46.89 | 12 Sep 2023 | 18.3 | 5.6 | 11.7 | 18.80 | 12.81 | 0.0 |
| Fargo, ND | 46.89 | 13 Sep 2023 | 22.2 | 7.8 | 15.0 | 19.72 | 12.75 | 0.0 |
| Fargo, ND | 46.89 | 14 Sep 2023 | 23.3 | 12.8 | 18.3 | 10.34 | 12.70 | 0.0 |
| Fargo, ND | 46.89 | 15 Sep 2023 | 22.8 | 11.1 | 17.2 | 19.01 | 12.64 | 0.0 |
| Fargo, ND | 46.89 | 16 Sep 2023 | 17.8 | 7.2 | 12.8 | 8.75 | 12.59 | 0.0 |
| Fargo, ND | 46.89 | 17 Sep 2023 | 22.2 | 4.4 | 13.3 | 19.01 | 12.53 | 0.0 |
| Fargo, ND | 46.89 | 18 Sep 2023 | 27.8 | 11.7 | 20.0 | 17.33 | 12.48 | 0.0 |
| Fargo, ND | 46.89 | 19 Sep 2023 | 29.4 | 16.1 | 22.8 | 15.91 | 12.42 | 0.0 |
| Fargo, ND | 46.89 | 20 Sep 2023 | 26.7 | 11.1 | 18.9 | 17.79 | 12.37 | 0.0 |
| Fargo, ND | 46.89 | 21 Sep 2023 | 27.2 | 17.2 | 22.2 | 9.92 | 12.31 | 0.0 |
| Fargo, ND | 46.89 | 22 Sep 2023 | 23.9 | 15.6 | 19.4 | 6.07 | 12.25 | 0.0 |
| Fargo, ND | 46.89 | 23 Sep 2023 | 21.7 | 15.0 | 18.3 | 3.31 | 12.20 | 0.0 |
| Fargo, ND | 46.89 | 24 Sep 2023 | 19.4 | 12.2 | 15.6 | 8.42 | 12.14 | 0.0 |
| Fargo, ND | 46.89 | 25 Sep 2023 | 20.6 | 11.1 | 16.1 | 8.79 | 12.09 | 0.0 |
| Fargo, ND | 46.89 | 26 Sep 2023 | 23.3 | 11.7 | 17.2 | 13.10 | 12.03 | 0.0 |
| Fargo, ND | 46.89 | 27 Sep 2023 | 20.6 | 14.4 | 17.8 | 7.75 | 11.98 | 0.0 |
| Fargo, ND | 46.89 | 28 Sep 2023 | 24.4 | 15.6 | 20.0 | 13.10 | 11.92 | 0.0 |
| Fargo, ND | 46.89 | 29 Sep 2023 | 22.8 | 12.2 | 17.8 | 12.18 | 11.87 | 0.0 |
| Fargo, ND | 46.89 | 30 Sep 2023 | 23.9 | 11.1 | 17.2 | 8.67 | 11.81 | 0.0 |
| Fargo, ND | 46.89 | 1 Oct 2023 | 33.9 | 19.4 | 26.7 | 15.03 | 11.75 | 0.0 |
| Fargo, ND | 46.89 | 2 Oct 2023 | 31.1 | 19.4 | 25.0 | 15.07 | 11.70 | 0.0 |
| Fargo, ND | 46.89 | 3 Oct 2023 | 25.6 | 16.1 | 21.1 | 9.59 | 11.64 | 0.0 |
| Fargo, ND | 46.89 | 4 Oct 2023 | 18.9 | 11.7 | 15.0 | 12.43 | 11.59 | 0.0 |
| Fargo, ND | 46.89 | 5 Oct 2023 | 13.9 | 6.1 | 10.0 | 6.74 | 11.53 | 0.0 |
| Fargo, ND | 46.89 | 6 Oct 2023 | 10.6 | 5.0 | 7.8 | 6.91 | 11.48 | 0.0 |
| Fargo, ND | 46.89 | 7 Oct 2023 | 12.8 | 0.6 | 6.7 | 9.04 | 11.42 | 0.0 |
| Fargo, ND | 46.89 | 8 Oct 2023 | 15.0 | 0.0 | 7.8 | 14.78 | 11.37 | 0.0 |
| Fargo, ND | 46.89 | 9 Oct 2023 | 12.2 | 0.6 | 6.7 | 11.51 | 11.31 | 0.0 |
| Fargo, ND | 46.89 | 10 Oct 2023 | 12.8 | -2.8 | 5.0 | 15.03 | 11.26 | 0.0 |
| Fargo, ND | 46.89 | 11 Oct 2023 | 16.1 | -1.1 | 7.2 | 14.15 | 11.20 | 0.0 |
| Fargo, ND | 46.89 | 12 Oct 2023 | 15.0 | 8.3 | 11.7 | 11.60 | 11.15 | 0.0 |
| Fargo, ND | 46.89 | 13 Oct 2023 | 11.1 | 6.7 | 8.9 | 4.27 | 11.09 | 0.0 |
| Fargo, ND | 46.89 | 14 Oct 2023 | 11.1 | 0.6 | 6.1 | 4.27 | 11.04 | 0.0 |
| Fargo, ND | 46.89 | 15 Oct 2023 | 14.4 | 2.2 | 8.3 | 13.31 | 10.98 | 0.0 |
| Fargo, ND | 46.89 | 16 Oct 2023 | 16.1 | 0.0 | 8.3 | 12.85 | 10.93 | 0.0 |
| Fargo, ND | 46.89 | 17 Oct 2023 | 20.0 | 4.4 | 12.2 | 9.84 | 10.88 | 0.0 |
| Fargo, ND | 46.89 | 18 Oct 2023 | 14.4 | 8.9 | 11.7 | 3.52 | 10.82 | 0.0 |
| Fargo, ND | 46.89 | 19 Oct 2023 | 17.8 | 5.6 | 11.7 | 10.84 | 10.77 | 0.0 |
| Fargo, ND | 46.89 | 20 Oct 2023 | 21.7 | 5.0 | 13.3 | 10.68 | 10.71 | 0.0 |
| Fargo, ND | 46.89 | 21 Oct 2023 | 13.9 | 5.6 | 9.4 | 7.08 | 10.66 | 0.0 |
| Fargo, ND | 46.89 | 22 Oct 2023 | 15.6 | 6.1 | 11.1 | 8.33 | 10.61 | 0.0 |
| Fargo, ND | 46.89 | 23 Oct 2023 | 17.2 | 7.8 | 12.8 | 11.14 | 10.55 | 0.0 |
| Fargo, ND | 46.89 | 24 Oct 2023 | 7.8 | 3.3 | 5.6 | 2.89 | 10.50 | 0.0 |
| Fargo, ND | 46.89 | 25 Oct 2023 | 8.9 | 1.7 | 5.6 | 6.24 | 10.45 | 0.0 |
| Fargo, ND | 46.89 | 26 Oct 2023 | 6.1 | 0.0 | 2.8 | 2.22 | 10.40 | 2.2 |
| Fargo, ND | 46.89 | 27 Oct 2023 | 0.0 | -6.7 | -3.3 | 4.56 | 10.34 | 8.3 |
| Fargo, ND | 46.89 | 28 Oct 2023 | -1.1 | -9.4 | -5.6 | 10.30 | 10.29 | 10.6 |
| Fargo, ND | 46.89 | 29 Oct 2023 | -2.2 | -7.8 | -5.0 | 9.96 | 10.24 | 10.0 |
| Fargo, ND | 46.89 | 30 Oct 2023 | 0.6 | -6.7 | -3.3 | 5.19 | 10.19 | 8.3 |
| Fargo, ND | 46.89 | 31 Oct 2023 | -3.3 | -5.6 | -4.4 | 6.82 | 10.14 | 9.4 |
| Fargo, ND | 46.89 | 1 Nov 2023 | 1.7 | -3.9 | -1.1 | 7.66 | 10.09 | 6.1 |
| Fargo, ND | 46.89 | 2 Nov 2023 | 3.9 | -6.7 | -1.7 | 5.69 | 10.04 | 6.7 |
| Fargo, ND | 46.89 | 3 Nov 2023 | 1.7 | -5.6 | -1.7 | 8.37 | 9.99 | 6.7 |
| Fargo, ND | 46.89 | 4 Nov 2023 | 5.0 | -4.4 | 0.6 | 5.32 | 9.94 | 4.4 |
| Fargo, ND | 46.89 | 5 Nov 2023 | 10.0 | 0.0 | 5.0 | 3.27 | 9.89 | 0.0 |
| Fargo, ND | 46.89 | 6 Nov 2023 | 7.8 | 0.0 | 3.9 | 2.26 | 9.84 | 1.1 |
| Fargo, ND | 46.89 | 7 Nov 2023 | 5.0 | 0.0 | 2.8 | 1.67 | 9.79 | 2.2 |
| Fargo, ND | 46.89 | 8 Nov 2023 | 7.8 | 0.6 | 4.4 | 3.85 | 9.75 | 0.6 |
| Fargo, ND | 46.89 | 9 Nov 2023 | 2.8 | 0.0 | 1.7 | 3.77 | 9.70 | 3.3 |
| Fargo, ND | 46.89 | 10 Nov 2023 | 1.7 | -0.6 | 0.6 | 2.76 | 9.65 | 4.4 |
| Fargo, ND | 46.89 | 11 Nov 2023 | 5.0 | -0.6 | 2.2 | 6.41 | 9.61 | 2.8 |
| Fargo, ND | 46.89 | 12 Nov 2023 | 13.3 | -3.9 | 5.0 | 8.29 | 9.56 | 0.0 |
| Fargo, ND | 46.89 | 13 Nov 2023 | 10.6 | -2.2 | 3.9 | 8.16 | 9.52 | 1.1 |
| Fargo, ND | 46.89 | 14 Nov 2023 | 14.4 | 0.6 | 7.2 | 7.49 | 9.47 | 0.0 |
| Fargo, ND | 46.89 | 15 Nov 2023 | 13.9 | -1.1 | 6.7 | 7.95 | 9.43 | 0.0 |
| Fargo, ND | 46.89 | 16 Nov 2023 | 5.6 | -5.6 | 0.0 | 6.15 | 9.38 | 5.0 |
| Fargo, ND | 46.89 | 17 Nov 2023 | 8.9 | -6.1 | 1.7 | 7.58 | 9.34 | 3.3 |
| Fargo, ND | 46.89 | 18 Nov 2023 | 11.1 | -1.7 | 4.4 | 7.91 | 9.30 | 0.6 |
| Fargo, ND | 46.89 | 19 Nov 2023 | 12.8 | -3.9 | 4.4 | 7.08 | 9.26 | 0.6 |
| Fargo, ND | 46.89 | 20 Nov 2023 | 9.4 | 1.1 | 5.6 | 3.73 | 9.22 | 0.0 |
| Fargo, ND | 46.89 | 21 Nov 2023 | 2.2 | -3.9 | -0.6 | 7.58 | 9.18 | 5.6 |
| Fargo, ND | 46.89 | 22 Nov 2023 | 7.2 | -6.1 | 0.6 | 5.11 | 9.14 | 4.4 |
| Fargo, ND | 46.89 | 23 Nov 2023 | -6.1 | -13.9 | -10.0 | 7.83 | 9.11 | 15.0 |
| Fargo, ND | 46.89 | 24 Nov 2023 | -1.7 | -14.4 | -7.8 | 7.49 | 9.07 | 12.8 |
| Fargo, ND | 46.89 | 25 Nov 2023 | -0.6 | -8.9 | -4.4 | 5.86 | 9.03 | 9.4 |
| Fargo, ND | 46.89 | 26 Nov 2023 | -0.6 | -15.6 | -7.8 | 4.14 | 9.00 | 12.8 |
| Fargo, ND | 46.89 | 27 Nov 2023 | -9.4 | -17.2 | -13.3 | 3.68 | 8.97 | 18.3 |
| Fargo, ND | 46.89 | 28 Nov 2023 | 4.4 | -14.4 | -5.0 | 6.78 | 8.93 | 10.0 |
| Fargo, ND | 46.89 | 29 Nov 2023 | 8.3 | -4.4 | 2.2 | 7.29 | 8.90 | 2.8 |
| Fargo, ND | 46.89 | 30 Nov 2023 | 2.2 | -7.2 | -2.8 | 4.98 | 8.87 | 7.8 |
| Fargo, ND | 46.89 | 1 Dec 2023 | 1.7 | -7.8 | -2.8 | 6.15 | 8.84 | 7.8 |
| Fargo, ND | 46.89 | 2 Dec 2023 | 3.3 | -5.0 | -0.6 | 5.82 | 8.81 | 5.6 |
| Fargo, ND | 46.89 | 3 Dec 2023 | 1.1 | -5.6 | -2.2 | 1.67 | 8.79 | 7.2 |
| Fargo, ND | 46.89 | 4 Dec 2023 | 1.1 | -6.1 | -2.2 | 4.02 | 8.76 | 7.2 |
| Fargo, ND | 46.89 | 5 Dec 2023 | -2.2 | -5.6 | -3.9 | 3.06 | 8.74 | 8.9 |
| Fargo, ND | 46.89 | 6 Dec 2023 | 11.1 | -3.3 | 3.9 | 4.48 | 8.71 | 1.1 |
| Fargo, ND | 46.89 | 7 Dec 2023 | 15.0 | -3.3 | 5.6 | 6.24 | 8.69 | 0.0 |
| Fargo, ND | 46.89 | 8 Dec 2023 | 5.0 | 0.0 | 2.8 | 1.00 | 8.67 | 2.2 |
| Fargo, ND | 46.89 | 9 Dec 2023 | 0.0 | -5.6 | -2.8 | 2.64 | 8.65 | 7.8 |
| Fargo, ND | 46.89 | 10 Dec 2023 | -5.0 | -7.8 | -6.1 | 2.26 | 8.63 | 11.1 |
| Fargo, ND | 46.89 | 11 Dec 2023 | -1.1 | -6.1 | -3.9 | 3.85 | 8.61 | 8.9 |
| Fargo, ND | 46.89 | 12 Dec 2023 | -1.7 | -13.3 | -7.8 | 6.41 | 8.60 | 12.8 |
| Fargo, ND | 46.89 | 13 Dec 2023 | 3.3 | -7.8 | -2.2 | 6.41 | 8.58 | 7.2 |
| Fargo, ND | 46.89 | 14 Dec 2023 | 9.4 | -2.2 | 3.9 | 5.95 | 8.57 | 1.1 |
| Fargo, ND | 46.89 | 15 Dec 2023 | 4.4 | 1.1 | 2.8 | 0.84 | 8.56 | 2.2 |
| Fargo, ND | 46.89 | 16 Dec 2023 | 1.1 | -2.8 | -1.1 | 2.05 | 8.55 | 6.1 |
| Fargo, ND | 46.89 | 17 Dec 2023 | 2.8 | -6.7 | -2.2 | 3.77 | 8.54 | 7.2 |
| Fargo, ND | 46.89 | 18 Dec 2023 | -6.1 | -15.6 | -10.6 | 6.20 | 8.53 | 15.6 |
| Fargo, ND | 46.89 | 19 Dec 2023 | -0.6 | -6.1 | -3.3 | 3.39 | 8.53 | 8.3 |
| Fargo, ND | 46.89 | 20 Dec 2023 | 3.9 | -2.8 | 0.6 | 5.28 | 8.52 | 4.4 |
| Fargo, ND | 46.89 | 21 Dec 2023 | 3.9 | 0.0 | 1.7 | 4.10 | 8.52 | 3.3 |
| Fargo, ND | 46.89 | 22 Dec 2023 | 2.2 | 0.0 | 1.1 | 2.43 | 8.52 | 3.9 |
| Fargo, ND | 46.89 | 23 Dec 2023 | 6.1 | 1.1 | 3.9 | 3.14 | 8.52 | 1.1 |
| Fargo, ND | 46.89 | 24 Dec 2023 | 6.1 | -2.8 | 1.7 | 0.84 | 8.52 | 3.3 |
| Fargo, ND | 46.89 | 25 Dec 2023 | 0.6 | -5.6 | -2.8 | 1.38 | 8.52 | 7.8 |
| Fargo, ND | 46.89 | 26 Dec 2023 | 1.1 | 0.0 | 0.6 | 1.13 | 8.53 | 4.4 |
| Fargo, ND | 46.89 | 27 Dec 2023 | 0.0 | -0.6 | 0.0 | 1.55 | 8.53 | 5.0 |
| Fargo, ND | 46.89 | 28 Dec 2023 | 0.0 | -5.0 | -2.2 | 3.31 | 8.54 | 7.2 |
| Fargo, ND | 46.89 | 29 Dec 2023 | 2.8 | -5.6 | -1.1 | 6.28 | 8.55 | 6.1 |
| Fargo, ND | 46.89 | 30 Dec 2023 | -1.7 | -5.6 | -3.9 | 1.13 | 8.56 | 8.9 |
| Fargo, ND | 46.89 | 31 Dec 2023 | -5.6 | -8.9 | -7.2 | 1.93 | 8.57 | 12.2 |
| Fargo, ND | 46.89 | 1 Jan 2024 | 0.6 | -8.3 | -3.9 | 6.11 | 8.59 | 8.9 |
| Fargo, ND | 46.89 | 2 Jan 2024 | 1.1 | -4.4 | -1.7 | 4.23 | 8.60 | 6.7 |
| Fargo, ND | 46.89 | 3 Jan 2024 | -2.2 | -9.4 | -5.6 | 3.10 | 8.62 | 10.6 |
| Fargo, ND | 46.89 | 4 Jan 2024 | -3.9 | -9.4 | -6.7 | 2.81 | 8.64 | 11.7 |
| Fargo, ND | 46.89 | 5 Jan 2024 | 0.0 | -3.9 | -1.7 | 0.92 | 8.66 | 6.7 |
| Fargo, ND | 46.89 | 6 Jan 2024 | 0.0 | -15.0 | -7.2 | 4.27 | 8.68 | 12.2 |
| Fargo, ND | 46.89 | 7 Jan 2024 | -13.9 | -16.1 | -15.0 | 4.90 | 8.70 | 20.0 |
| Fargo, ND | 46.89 | 8 Jan 2024 | -10.0 | -15.6 | -12.8 | 4.52 | 8.72 | 17.8 |
| Fargo, ND | 46.89 | 9 Jan 2024 | -11.1 | -16.1 | -13.3 | 6.78 | 8.74 | 18.3 |
| Fargo, ND | 46.89 | 10 Jan 2024 | -6.1 | -14.4 | -10.0 | 2.55 | 8.77 | 15.0 |
| Fargo, ND | 46.89 | 11 Jan 2024 | -14.4 | -20.6 | -17.2 | 5.28 | 8.80 | 22.2 |
| Fargo, ND | 46.89 | 12 Jan 2024 | -17.2 | -21.7 | -19.4 | 3.64 | 8.82 | 24.4 |
| Fargo, ND | 46.89 | 13 Jan 2024 | -18.9 | -23.3 | -21.1 | 3.60 | 8.85 | 26.1 |
| Fargo, ND | 46.89 | 14 Jan 2024 | -21.7 | -27.2 | -24.4 | 6.66 | 8.88 | 29.4 |
| Fargo, ND | 46.89 | 15 Jan 2024 | -18.9 | -25.6 | -22.2 | 5.82 | 8.91 | 27.2 |
| Fargo, ND | 46.89 | 16 Jan 2024 | -13.3 | -20.6 | -17.2 | 6.49 | 8.95 | 22.2 |
| Fargo, ND | 46.89 | 17 Jan 2024 | -14.4 | -20.6 | -17.8 | 5.82 | 8.98 | 22.8 |
| Fargo, ND | 46.89 | 18 Jan 2024 | -16.1 | -21.1 | -18.3 | 6.32 | 9.01 | 23.3 |
| Fargo, ND | 46.89 | 19 Jan 2024 | -16.1 | -23.3 | -19.4 | 7.87 | 9.05 | 24.4 |
| Fargo, ND | 46.89 | 20 Jan 2024 | -13.3 | -21.7 | -17.8 | 7.79 | 9.09 | 22.8 |
| Fargo, ND | 46.89 | 21 Jan 2024 | -5.6 | -15.0 | -10.0 | 4.40 | 9.12 | 15.0 |
| Fargo, ND | 46.89 | 22 Jan 2024 | -5.6 | -8.3 | -6.7 | 3.27 | 9.16 | 11.7 |
| Fargo, ND | 46.89 | 23 Jan 2024 | -0.6 | -8.3 | -4.4 | 3.31 | 9.20 | 9.4 |
| Fargo, ND | 46.89 | 24 Jan 2024 | -0.6 | -1.7 | -1.1 | 1.51 | 9.24 | 6.1 |
| Fargo, ND | 46.89 | 25 Jan 2024 | -1.1 | -1.7 | -1.7 | 1.67 | 9.28 | 6.7 |
| Fargo, ND | 46.89 | 26 Jan 2024 | -1.7 | -3.3 | -2.2 | 2.47 | 9.32 | 7.2 |
| Fargo, ND | 46.89 | 27 Jan 2024 | 2.8 | -7.2 | -2.2 | 8.62 | 9.36 | 7.2 |
| Fargo, ND | 46.89 | 28 Jan 2024 | 5.6 | -4.4 | 0.6 | 8.75 | 9.41 | 4.4 |
| Fargo, ND | 46.89 | 29 Jan 2024 | 5.6 | -1.1 | 2.2 | 7.70 | 9.45 | 2.8 |
| Fargo, ND | 46.89 | 30 Jan 2024 | 3.9 | -1.1 | 1.1 | 4.40 | 9.49 | 3.9 |
| Fargo, ND | 46.89 | 31 Jan 2024 | 10.0 | -2.2 | 3.9 | 8.67 | 9.54 | 1.1 |
| Fargo, ND | 46.89 | 1 Feb 2024 | 6.7 | -3.3 | 1.7 | 8.83 | 9.58 | 3.3 |
| Fargo, ND | 46.89 | 2 Feb 2024 | 6.1 | -1.7 | 2.2 | 9.04 | 9.63 | 2.8 |
| Fargo, ND | 46.89 | 3 Feb 2024 | 2.2 | -0.6 | 1.1 | 2.47 | 9.68 | 3.9 |
| Fargo, ND | 46.89 | 4 Feb 2024 | 0.6 | -1.1 | 0.0 | 1.84 | 9.72 | 5.0 |
| Fargo, ND | 46.89 | 5 Feb 2024 | 1.7 | -1.1 | 0.0 | 2.39 | 9.77 | 5.0 |
| Fargo, ND | 46.89 | 6 Feb 2024 | 3.9 | 0.0 | 2.2 | 3.35 | 9.82 | 2.8 |
| Fargo, ND | 46.89 | 7 Feb 2024 | 8.9 | 1.1 | 5.0 | 4.65 | 9.87 | 0.0 |
| Fargo, ND | 46.89 | 8 Feb 2024 | 8.3 | -3.9 | 2.2 | 1.72 | 9.92 | 2.8 |
| Fargo, ND | 46.89 | 9 Feb 2024 | -2.2 | -6.7 | -4.4 | 5.28 | 9.97 | 9.4 |
| Fargo, ND | 46.89 | 10 Feb 2024 | -0.6 | -7.8 | -3.9 | 4.48 | 10.02 | 8.9 |
| Fargo, ND | 46.89 | 11 Feb 2024 | 2.8 | -10.0 | -3.3 | 9.42 | 10.07 | 8.3 |
| Fargo, ND | 46.89 | 12 Feb 2024 | 1.1 | -7.2 | -3.3 | 10.97 | 10.12 | 8.3 |
| Fargo, ND | 46.89 | 13 Feb 2024 | 1.7 | -2.2 | -0.6 | 6.70 | 10.17 | 5.6 |
| Fargo, ND | 46.89 | 14 Feb 2024 | -0.6 | -7.2 | -3.9 | 7.58 | 10.22 | 8.9 |
| Fargo, ND | 46.89 | 15 Feb 2024 | -6.1 | -11.1 | -8.9 | 10.26 | 10.28 | 13.9 |
| Fargo, ND | 46.89 | 16 Feb 2024 | -6.7 | -13.9 | -10.6 | 9.92 | 10.33 | 15.6 |
| Fargo, ND | 46.89 | 17 Feb 2024 | 3.9 | -9.4 | -2.8 | 11.97 | 10.38 | 7.8 |
| Fargo, ND | 46.89 | 18 Feb 2024 | 0.6 | -7.8 | -3.3 | 12.31 | 10.43 | 8.3 |
| Fargo, ND | 46.89 | 19 Feb 2024 | 6.7 | -4.4 | 1.1 | 12.64 | 10.49 | 3.9 |
| Fargo, ND | 46.89 | 20 Feb 2024 | 3.9 | -5.0 | -0.6 | 11.64 | 10.54 | 5.6 |
| Fargo, ND | 46.89 | 21 Feb 2024 | 8.3 | -2.8 | 2.8 | 10.38 | 10.59 | 2.2 |
| Fargo, ND | 46.89 | 22 Feb 2024 | 7.8 | -2.8 | 2.8 | 11.72 | 10.65 | 2.2 |
| Fargo, ND | 46.89 | 23 Feb 2024 | 0.0 | -6.7 | -3.3 | 8.62 | 10.70 | 8.3 |
| Fargo, ND | 46.89 | 24 Feb 2024 | 11.1 | -6.7 | 2.2 | 12.48 | 10.76 | 2.8 |
| Fargo, ND | 46.89 | 25 Feb 2024 | 2.8 | -1.7 | 0.6 | 10.93 | 10.81 | 4.4 |
| Fargo, ND | 46.89 | 26 Feb 2024 | 14.4 | -1.7 | 6.1 | 11.85 | 10.87 | 0.0 |
| Fargo, ND | 46.89 | 27 Feb 2024 | -1.7 | -18.9 | -10.6 | 7.87 | 10.92 | 15.6 |
| Fargo, ND | 46.89 | 28 Feb 2024 | -12.2 | -23.9 | -17.8 | 14.99 | 10.98 | 22.8 |
| Fargo, ND | 46.89 | 29 Feb 2024 | 6.1 | -13.3 | -3.9 | 14.57 | 11.03 | 8.9 |
| Parlier, CA | 36.60 | 1 Sep 2023 | 34.4 | 17.8 | 25.8 | 23.98 | 13.00 | 0.0 |
| Parlier, CA | 36.60 | 2 Sep 2023 | 30.3 | 19.6 | 23.8 | 17.42 | 12.96 | 0.0 |
| Parlier, CA | 36.60 | 3 Sep 2023 | 25.9 | 19.8 | 22.5 | 18.77 | 12.93 | 0.0 |
| Parlier, CA | 36.60 | 4 Sep 2023 | 29.4 | 16.1 | 22.1 | 23.28 | 12.89 | 0.0 |
| Parlier, CA | 36.60 | 5 Sep 2023 | 33.2 | 17.5 | 24.6 | 23.16 | 12.85 | 0.0 |
| Parlier, CA | 36.60 | 6 Sep 2023 | 34.0 | 18.5 | 25.6 | 23.00 | 12.81 | 0.0 |
| Parlier, CA | 36.60 | 7 Sep 2023 | 34.6 | 19.1 | 26.1 | 22.95 | 12.78 | 0.0 |
| Parlier, CA | 36.60 | 8 Sep 2023 | 37.3 | 18.2 | 26.8 | 22.77 | 12.74 | 0.0 |
| Parlier, CA | 36.60 | 9 Sep 2023 | 37.3 | 22.0 | 28.8 | 21.51 | 12.70 | 0.0 |
| Parlier, CA | 36.60 | 10 Sep 2023 | 34.7 | 21.2 | 27.7 | 19.14 | 12.66 | 0.0 |
| Parlier, CA | 36.60 | 11 Sep 2023 | 34.9 | 21.1 | 27.6 | 17.18 | 12.62 | 0.0 |
| Parlier, CA | 36.60 | 12 Sep 2023 | 35.0 | 18.4 | 26.1 | 22.38 | 12.59 | 0.0 |
| Parlier, CA | 36.60 | 13 Sep 2023 | 34.4 | 17.6 | 25.3 | 22.24 | 12.55 | 0.0 |
| Parlier, CA | 36.60 | 14 Sep 2023 | 35.3 | 18.5 | 26.4 | 21.60 | 12.51 | 0.0 |
| Parlier, CA | 36.60 | 15 Sep 2023 | 36.5 | 19.6 | 27.4 | 21.32 | 12.47 | 0.0 |
| Parlier, CA | 36.60 | 16 Sep 2023 | 34.7 | 20.4 | 27.0 | 21.12 | 12.43 | 0.0 |
| Parlier, CA | 36.60 | 17 Sep 2023 | 31.1 | 18.7 | 24.5 | 20.56 | 12.39 | 0.0 |
| Parlier, CA | 36.60 | 18 Sep 2023 | 30.4 | 17.8 | 23.3 | 20.80 | 12.36 | 0.0 |
| Parlier, CA | 36.60 | 19 Sep 2023 | 31.1 | 17.6 | 23.7 | 20.83 | 12.32 | 0.0 |
| Parlier, CA | 36.60 | 20 Sep 2023 | 31.7 | 18.5 | 24.3 | 20.50 | 12.28 | 0.0 |
| Parlier, CA | 36.60 | 21 Sep 2023 | 28.5 | 16.9 | 22.0 | 20.52 | 12.24 | 0.0 |
| Parlier, CA | 36.60 | 22 Sep 2023 | 30.1 | 15.0 | 21.4 | 20.63 | 12.20 | 0.0 |
| Parlier, CA | 36.60 | 23 Sep 2023 | 31.5 | 16.5 | 22.7 | 20.32 | 12.16 | 0.0 |
| Parlier, CA | 36.60 | 24 Sep 2023 | 32.6 | 14.5 | 22.4 | 20.78 | 12.13 | 0.0 |
| Parlier, CA | 36.60 | 25 Sep 2023 | 31.6 | 13.9 | 21.8 | 20.28 | 12.09 | 0.0 |
| Parlier, CA | 36.60 | 26 Sep 2023 | 30.4 | 14.7 | 22.1 | 19.89 | 12.05 | 0.0 |
| Parlier, CA | 36.60 | 27 Sep 2023 | 29.8 | 15.8 | 22.2 | 19.66 | 12.01 | 0.0 |
| Parlier, CA | 36.60 | 28 Sep 2023 | 30.0 | 15.1 | 21.5 | 19.56 | 11.97 | 0.0 |
| Parlier, CA | 36.60 | 29 Sep 2023 | 30.7 | 14.8 | 22.2 | 19.50 | 11.93 | 0.0 |
| Parlier, CA | 36.60 | 30 Sep 2023 | 22.5 | 14.7 | 18.3 | 12.64 | 11.89 | 0.0 |
| Parlier, CA | 36.60 | 1 Oct 2023 | 23.5 | 12.8 | 17.3 | 16.59 | 11.85 | 0.0 |
| Parlier, CA | 36.60 | 2 Oct 2023 | 25.7 | 13.7 | 18.9 | 16.54 | 11.82 | 0.0 |
| Parlier, CA | 36.60 | 3 Oct 2023 | 27.2 | 13.0 | 19.6 | 18.46 | 11.78 | 0.0 |
| Parlier, CA | 36.60 | 4 Oct 2023 | 32.1 | 14.1 | 22.3 | 18.54 | 11.74 | 0.0 |
| Parlier, CA | 36.60 | 5 Oct 2023 | 33.8 | 17.0 | 24.0 | 18.37 | 11.70 | 0.0 |
| Parlier, CA | 36.60 | 6 Oct 2023 | 34.5 | 17.1 | 24.4 | 18.21 | 11.66 | 0.0 |
| Parlier, CA | 36.60 | 7 Oct 2023 | 35.3 | 18.1 | 24.9 | 18.13 | 11.62 | 0.0 |
| Parlier, CA | 36.60 | 8 Oct 2023 | 36.3 | 17.3 | 25.6 | 17.92 | 11.59 | 0.0 |
| Parlier, CA | 36.60 | 9 Oct 2023 | 31.1 | 15.7 | 22.0 | 14.50 | 11.55 | 0.0 |
| Parlier, CA | 36.60 | 10 Oct 2023 | 27.1 | 14.4 | 20.1 | 17.20 | 11.51 | 0.0 |
| Parlier, CA | 36.60 | 11 Oct 2023 | 23.6 | 14.5 | 18.4 | 17.36 | 11.47 | 0.0 |
| Parlier, CA | 36.60 | 12 Oct 2023 | 25.0 | 11.0 | 17.4 | 17.47 | 11.43 | 0.0 |
| Parlier, CA | 36.60 | 13 Oct 2023 | 28.9 | 11.2 | 18.7 | 16.65 | 11.40 | 0.0 |
| Parlier, CA | 36.60 | 14 Oct 2023 | 29.6 | 12.4 | 19.8 | 16.91 | 11.36 | 0.0 |
| Parlier, CA | 36.60 | 15 Oct 2023 | 30.2 | 12.1 | 20.0 | 16.66 | 11.32 | 0.0 |
| Parlier, CA | 36.60 | 16 Oct 2023 | 30.2 | 13.9 | 21.3 | 16.43 | 11.28 | 0.0 |
| Parlier, CA | 36.60 | 17 Oct 2023 | 27.2 | 13.4 | 19.5 | 16.48 | 11.25 | 0.0 |
| Parlier, CA | 36.60 | 18 Oct 2023 | 30.5 | 14.5 | 20.7 | 16.29 | 11.21 | 0.0 |
| Parlier, CA | 36.60 | 19 Oct 2023 | 31.8 | 14.6 | 22.3 | 16.18 | 11.17 | 0.0 |
| Parlier, CA | 36.60 | 20 Oct 2023 | 33.8 | 15.9 | 23.6 | 15.67 | 11.13 | 0.0 |
| Parlier, CA | 36.60 | 21 Oct 2023 | 28.5 | 14.9 | 20.8 | 15.38 | 11.10 | 0.0 |
| Parlier, CA | 36.60 | 22 Oct 2023 | 25.1 | 13.2 | 19.0 | 14.68 | 11.06 | 0.0 |
| Parlier, CA | 36.60 | 23 Oct 2023 | 22.2 | 14.8 | 17.4 | 10.83 | 11.02 | 0.0 |
| Parlier, CA | 36.60 | 24 Oct 2023 | 25.1 | 12.4 | 17.7 | 15.09 | 10.99 | 0.0 |
| Parlier, CA | 36.60 | 25 Oct 2023 | 26.2 | 13.0 | 18.6 | 14.88 | 10.95 | 0.0 |
| Parlier, CA | 36.60 | 26 Oct 2023 | 21.4 | 12.4 | 15.8 | 15.00 | 10.92 | 0.0 |
| Parlier, CA | 36.60 | 27 Oct 2023 | 21.1 | 8.7 | 14.3 | 14.14 | 10.88 | 0.0 |
| Parlier, CA | 36.60 | 28 Oct 2023 | 19.6 | 6.8 | 12.6 | 15.28 | 10.84 | 0.0 |
| Parlier, CA | 36.60 | 29 Oct 2023 | 21.4 | 6.3 | 12.7 | 15.04 | 10.81 | 0.0 |
| Parlier, CA | 36.60 | 30 Oct 2023 | 23.0 | 6.6 | 13.5 | 15.07 | 10.77 | 0.0 |
| Parlier, CA | 36.60 | 31 Oct 2023 | 23.7 | 7.0 | 13.9 | 14.60 | 10.74 | 0.0 |
| Parlier, CA | 36.60 | 1 Nov 2023 | 24.1 | 6.8 | 14.1 | 14.70 | 10.70 | 0.0 |
| Parlier, CA | 36.60 | 2 Nov 2023 | 25.2 | 9.3 | 15.3 | 14.32 | 10.67 | 0.0 |
| Parlier, CA | 36.60 | 3 Nov 2023 | 24.3 | 9.3 | 15.3 | 13.68 | 10.64 | 0.0 |
| Parlier, CA | 36.60 | 4 Nov 2023 | 25.7 | 10.9 | 16.6 | 13.08 | 10.60 | 0.0 |
| Parlier, CA | 36.60 | 5 Nov 2023 | 25.7 | 10.0 | 16.9 | 12.84 | 10.57 | 0.0 |
| Parlier, CA | 36.60 | 6 Nov 2023 | 24.7 | 11.4 | 17.4 | 12.58 | 10.54 | 0.0 |
| Parlier, CA | 36.60 | 7 Nov 2023 | 19.7 | 11.7 | 15.2 | 12.93 | 10.50 | 0.0 |
| Parlier, CA | 36.60 | 8 Nov 2023 | 19.3 | 8.1 | 12.9 | 13.20 | 10.47 | 0.0 |
| Parlier, CA | 36.60 | 9 Nov 2023 | 19.8 | 5.8 | 12.3 | 13.28 | 10.44 | 0.0 |
| Parlier, CA | 36.60 | 10 Nov 2023 | 19.0 | 6.8 | 11.6 | 12.54 | 10.41 | 0.0 |
| Parlier, CA | 36.60 | 11 Nov 2023 | 19.8 | 6.2 | 11.8 | 12.86 | 10.38 | 0.0 |
| Parlier, CA | 36.60 | 12 Nov 2023 | 22.9 | 7.7 | 13.5 | 13.06 | 10.35 | 0.0 |
| Parlier, CA | 36.60 | 13 Nov 2023 | 23.3 | 9.3 | 14.6 | 10.69 | 10.32 | 0.0 |
| Parlier, CA | 36.60 | 14 Nov 2023 | 22.1 | 7.5 | 13.9 | 12.02 | 10.29 | 0.0 |
| Parlier, CA | 36.60 | 15 Nov 2023 | 22.7 | 7.9 | 13.5 | 4.95 | 10.26 | 0.0 |
| Parlier, CA | 36.60 | 16 Nov 2023 | 22.2 | 10.4 | 15.6 | 11.14 | 10.23 | 0.0 |
| Parlier, CA | 36.60 | 17 Nov 2023 | 22.1 | 9.4 | 15.4 | 10.45 | 10.20 | 0.0 |
| Parlier, CA | 36.60 | 18 Nov 2023 | 19.3 | 11.7 | 15.0 | 5.95 | 10.17 | 0.0 |
| Parlier, CA | 36.60 | 19 Nov 2023 | 16.1 | 8.9 | 12.2 | 11.38 | 10.14 | 0.0 |
| Parlier, CA | 36.60 | 20 Nov 2023 | 16.3 | 5.5 | 9.8 | 11.91 | 10.12 | 0.0 |
| Parlier, CA | 36.60 | 21 Nov 2023 | 20.3 | 5.9 | 11.7 | 11.87 | 10.09 | 0.0 |
| Parlier, CA | 36.60 | 22 Nov 2023 | 19.1 | 5.3 | 11.5 | 11.45 | 10.07 | 0.0 |
| Parlier, CA | 36.60 | 23 Nov 2023 | 18.5 | 7.3 | 12.5 | 10.99 | 10.04 | 0.0 |
| Parlier, CA | 36.60 | 24 Nov 2023 | 16.1 | 5.7 | 9.7 | 11.46 | 10.02 | 0.0 |
| Parlier, CA | 36.60 | 25 Nov 2023 | 16.5 | 3.5 | 8.7 | 11.44 | 9.99 | 0.0 |
| Parlier, CA | 36.60 | 26 Nov 2023 | 16.6 | 2.7 | 8.7 | 10.79 | 9.97 | 0.0 |
| Parlier, CA | 36.60 | 27 Nov 2023 | 17.2 | 2.4 | 8.5 | 11.46 | 9.95 | 0.0 |
| Parlier, CA | 36.60 | 28 Nov 2023 | 18.7 | 3.6 | 9.7 | 10.97 | 9.93 | 0.0 |
| Parlier, CA | 36.60 | 29 Nov 2023 | 17.6 | 4.0 | 9.8 | 10.71 | 9.91 | 0.0 |
| Parlier, CA | 36.60 | 30 Nov 2023 | 17.1 | 4.0 | 9.4 | 9.77 | 9.89 | 0.0 |
| Parlier, CA | 36.60 | 1 Dec 2023 | 15.6 | 5.4 | 10.0 | 10.68 | 9.87 | 0.0 |
| Parlier, CA | 36.60 | 2 Dec 2023 | 16.4 | 3.8 | 9.4 | 10.20 | 9.85 | 0.0 |
| Parlier, CA | 36.60 | 3 Dec 2023 | 15.9 | 4.9 | 9.6 | 7.70 | 9.83 | 0.0 |
| Parlier, CA | 36.60 | 4 Dec 2023 | 17.9 | 6.3 | 11.1 | 9.41 | 9.81 | 0.0 |
| Parlier, CA | 36.60 | 5 Dec 2023 | 18.9 | 4.9 | 11.5 | 10.45 | 9.80 | 0.0 |
| Parlier, CA | 36.60 | 6 Dec 2023 | 23.8 | 6.2 | 13.4 | 10.32 | 9.78 | 0.0 |
| Parlier, CA | 36.60 | 7 Dec 2023 | 17.6 | 8.7 | 12.4 | 10.35 | 9.77 | 0.0 |
| Parlier, CA | 36.60 | 8 Dec 2023 | 11.9 | 4.8 | 7.9 | 5.50 | 9.75 | 0.0 |
| Parlier, CA | 36.60 | 9 Dec 2023 | 15.4 | 3.4 | 7.8 | 10.20 | 9.74 | 0.0 |
| Parlier, CA | 36.60 | 10 Dec 2023 | 16.7 | 2.4 | 8.3 | 9.95 | 9.73 | 0.0 |
| Parlier, CA | 36.60 | 11 Dec 2023 | 15.9 | 2.9 | 8.5 | 10.21 | 9.72 | 0.0 |
| Parlier, CA | 36.60 | 12 Dec 2023 | 15.3 | 2.3 | 8.8 | 10.40 | 9.71 | 0.0 |
| Parlier, CA | 36.60 | 13 Dec 2023 | 17.0 | 2.9 | 8.5 | 10.41 | 9.70 | 0.0 |
| Parlier, CA | 36.60 | 14 Dec 2023 | 18.3 | 4.2 | 9.8 | 9.80 | 9.69 | 0.0 |
| Parlier, CA | 36.60 | 15 Dec 2023 | 18.7 | 4.4 | 10.7 | 9.55 | 9.68 | 0.0 |
| Parlier, CA | 36.60 | 16 Dec 2023 | 20.1 | 5.5 | 11.5 | 10.44 | 9.67 | 0.0 |
| Parlier, CA | 36.60 | 17 Dec 2023 | 19.4 | 6.5 | 11.9 | 7.33 | 9.67 | 0.0 |
| Parlier, CA | 36.60 | 18 Dec 2023 | 20.3 | 8.5 | 13.1 | 5.97 | 9.66 | 0.0 |
| Parlier, CA | 36.60 | 19 Dec 2023 | 17.4 | 11.1 | 13.7 | 5.34 | 9.66 | 0.0 |
| Parlier, CA | 36.60 | 20 Dec 2023 | 18.8 | 10.1 | 13.3 | 5.03 | 9.66 | 0.0 |
| Parlier, CA | 36.60 | 21 Dec 2023 | 16.2 | 9.9 | 12.3 | 5.46 | 9.65 | 0.0 |
| Parlier, CA | 36.60 | 22 Dec 2023 | 14.3 | 7.7 | 11.1 | 3.96 | 9.65 | 0.0 |
| Parlier, CA | 36.60 | 23 Dec 2023 | 16.0 | 6.5 | 10.0 | 8.13 | 9.65 | 0.0 |
| Parlier, CA | 36.60 | 24 Dec 2023 | 14.4 | 5.1 | 9.3 | 8.40 | 9.65 | 0.0 |
| Parlier, CA | 36.60 | 25 Dec 2023 | 13.8 | 5.0 | 8.8 | 8.29 | 9.66 | 0.0 |
| Parlier, CA | 36.60 | 26 Dec 2023 | 17.3 | 4.8 | 10.1 | 8.19 | 9.66 | 0.0 |
| Parlier, CA | 36.60 | 27 Dec 2023 | 17.6 | 7.0 | 11.4 | 7.96 | 9.66 | 0.0 |
| Parlier, CA | 36.60 | 28 Dec 2023 | 19.1 | 6.4 | 11.7 | 9.73 | 9.67 | 0.0 |
| Parlier, CA | 36.60 | 29 Dec 2023 | 19.6 | 7.9 | 12.5 | 8.46 | 9.67 | 0.0 |
| Parlier, CA | 36.60 | 30 Dec 2023 | 11.9 | 7.6 | 10.5 | 2.12 | 9.68 | 0.0 |
| Parlier, CA | 36.60 | 31 Dec 2023 | 14.5 | 5.0 | 9.3 | 6.33 | 9.69 | 0.0 |
| Parlier, CA | 36.60 | 1 Jan 2024 | 13.7 | 5.2 | 8.8 | 8.51 | 9.70 | 0.0 |
| Parlier, CA | 36.60 | 2 Jan 2024 | 14.5 | 3.3 | 8.1 | 7.84 | 9.71 | 0.0 |
| Parlier, CA | 36.60 | 3 Jan 2024 | 12.4 | 5.7 | 8.9 | 8.02 | 9.72 | 0.0 |
| Parlier, CA | 36.60 | 4 Jan 2024 | 10.3 | 3.9 | 6.6 | 6.63 | 9.73 | 0.0 |
| Parlier, CA | 36.60 | 5 Jan 2024 | 12.6 | 3.0 | 6.9 | 10.45 | 9.74 | 0.0 |
| Parlier, CA | 36.60 | 6 Jan 2024 | 11.3 | 2.0 | 6.2 | 5.05 | 9.76 | 0.0 |
| Parlier, CA | 36.60 | 7 Jan 2024 | 7.4 | 2.2 | 4.6 | 8.34 | 9.77 | 0.4 |
| Parlier, CA | 36.60 | 8 Jan 2024 | 10.6 | -0.1 | 4.2 | 10.43 | 9.79 | 0.8 |
| Parlier, CA | 36.60 | 9 Jan 2024 | 12.0 | -0.1 | 5.4 | 9.88 | 9.80 | 0.0 |
| Parlier, CA | 36.60 | 10 Jan 2024 | 12.3 | 4.5 | 7.9 | 9.33 | 9.82 | 0.0 |
| Parlier, CA | 36.60 | 11 Jan 2024 | 9.9 | 2.0 | 6.2 | 8.39 | 9.84 | 0.0 |
| Parlier, CA | 36.60 | 12 Jan 2024 | 11.6 | -0.5 | 4.9 | 10.14 | 9.85 | 0.1 |
| Parlier, CA | 36.60 | 13 Jan 2024 | 8.1 | 0.8 | 4.6 | 3.38 | 9.87 | 0.4 |
| Parlier, CA | 36.60 | 14 Jan 2024 | 15.6 | 3.5 | 8.6 | 9.90 | 9.89 | 0.0 |
| Parlier, CA | 36.60 | 15 Jan 2024 | 14.1 | 4.5 | 8.2 | 7.02 | 9.91 | 0.0 |
| Parlier, CA | 36.60 | 16 Jan 2024 | 14.3 | 3.0 | 8.2 | 7.21 | 9.94 | 0.0 |
| Parlier, CA | 36.60 | 17 Jan 2024 | 17.5 | 7.3 | 11.5 | 10.81 | 9.96 | 0.0 |
| Parlier, CA | 36.60 | 18 Jan 2024 | 15.7 | 5.0 | 9.9 | 8.20 | 9.98 | 0.0 |
| Parlier, CA | 36.60 | 19 Jan 2024 | 18.7 | 6.8 | 11.5 | 7.52 | 10.00 | 0.0 |
| Parlier, CA | 36.60 | 20 Jan 2024 | 18.6 | 10.4 | 13.2 | 7.65 | 10.03 | 0.0 |
| Parlier, CA | 36.60 | 21 Jan 2024 | 17.6 | 10.8 | 13.0 | 9.72 | 10.05 | 0.0 |
| Parlier, CA | 36.60 | 22 Jan 2024 | 14.0 | 8.6 | 11.6 | 3.69 | 10.08 | 0.0 |
| Parlier, CA | 36.60 | 23 Jan 2024 | 16.5 | 6.9 | 11.2 | 8.24 | 10.10 | 0.0 |
| Parlier, CA | 36.60 | 24 Jan 2024 | 12.9 | 8.1 | 10.7 | 3.42 | 10.13 | 0.0 |
| Parlier, CA | 36.60 | 25 Jan 2024 | 17.1 | 7.0 | 11.7 | 11.01 | 10.16 | 0.0 |
| Parlier, CA | 36.60 | 26 Jan 2024 | 17.1 | 5.2 | 10.6 | 11.29 | 10.19 | 0.0 |
| Parlier, CA | 36.60 | 27 Jan 2024 | 17.2 | 7.0 | 11.4 | 9.03 | 10.21 | 0.0 |
| Parlier, CA | 36.60 | 28 Jan 2024 | 19.8 | 6.9 | 12.5 | 11.96 | 10.24 | 0.0 |
| Parlier, CA | 36.60 | 29 Jan 2024 | 22.5 | 8.3 | 14.1 | 12.47 | 10.27 | 0.0 |
| Parlier, CA | 36.60 | 30 Jan 2024 | 21.6 | 7.6 | 13.5 | 11.21 | 10.30 | 0.0 |
| Parlier, CA | 36.60 | 31 Jan 2024 | 20.1 | 9.9 | 14.6 | 7.19 | 10.33 | 0.0 |
| Parlier, CA | 36.60 | 1 Feb 2024 | 14.7 | 9.3 | 11.9 | 6.80 | 10.36 | 0.0 |
| Parlier, CA | 36.60 | 2 Feb 2024 | 14.3 | 6.2 | 9.2 | 9.68 | 10.39 | 0.0 |
| Parlier, CA | 36.60 | 3 Feb 2024 | 13.7 | 4.2 | 9.1 | 10.53 | 10.43 | 0.0 |
| Parlier, CA | 36.60 | 4 Feb 2024 | 18.7 | 8.2 | 12.3 | 7.25 | 10.46 | 0.0 |
| Parlier, CA | 36.60 | 5 Feb 2024 | 16.5 | 10.2 | 12.4 | 9.54 | 10.49 | 0.0 |
| Parlier, CA | 36.60 | 6 Feb 2024 | 12.5 | 6.8 | 10.3 | 7.26 | 10.52 | 0.0 |
| Parlier, CA | 36.60 | 7 Feb 2024 | 11.9 | 6.1 | 8.5 | 5.82 | 10.56 | 0.0 |
| Parlier, CA | 36.60 | 8 Feb 2024 | 11.1 | 3.4 | 6.5 | 8.76 | 10.59 | 0.0 |
| Parlier, CA | 36.60 | 9 Feb 2024 | 12.8 | 0.3 | 6.1 | 13.33 | 10.62 | 0.0 |
| Parlier, CA | 36.60 | 10 Feb 2024 | 13.2 | 2.1 | 6.8 | 13.99 | 10.66 | 0.0 |
| Parlier, CA | 36.60 | 11 Feb 2024 | 15.7 | 1.9 | 8.0 | 14.79 | 10.69 | 0.0 |
| Parlier, CA | 36.60 | 12 Feb 2024 | 16.3 | 2.6 | 8.6 | 15.11 | 10.73 | 0.0 |
| Parlier, CA | 36.60 | 13 Feb 2024 | 16.7 | 3.1 | 8.9 | 13.71 | 10.76 | 0.0 |
| Parlier, CA | 36.60 | 14 Feb 2024 | 16.5 | 4.4 | 9.9 | 11.01 | 10.80 | 0.0 |
| Parlier, CA | 36.60 | 15 Feb 2024 | 17.3 | 6.9 | 11.9 | 13.94 | 10.83 | 0.0 |
| Parlier, CA | 36.60 | 16 Feb 2024 | 17.4 | 6.6 | 11.1 | 11.85 | 10.87 | 0.0 |
| Parlier, CA | 36.60 | 17 Feb 2024 | 14.3 | 7.6 | 10.9 | 4.67 | 10.90 | 0.0 |
| Parlier, CA | 36.60 | 18 Feb 2024 | 20.7 | 7.1 | 13.3 | 15.50 | 10.94 | 0.0 |
| Parlier, CA | 36.60 | 19 Feb 2024 | 18.0 | 10.6 | 13.8 | 12.24 | 10.98 | 0.0 |
| Parlier, CA | 36.60 | 20 Feb 2024 | 18.8 | 7.4 | 12.5 | 14.67 | 11.01 | 0.0 |
| Parlier, CA | 36.60 | 21 Feb 2024 | 16.9 | 8.0 | 11.9 | 14.02 | 11.05 | 0.0 |
| Parlier, CA | 36.60 | 22 Feb 2024 | 17.4 | 5.4 | 10.5 | 16.61 | 11.09 | 0.0 |
| Parlier, CA | 36.60 | 23 Feb 2024 | 17.3 | 5.3 | 10.3 | 12.60 | 11.13 | 0.0 |
| Parlier, CA | 36.60 | 24 Feb 2024 | 20.4 | 5.4 | 11.8 | 15.49 | 11.16 | 0.0 |
| Parlier, CA | 36.60 | 25 Feb 2024 | 21.0 | 6.0 | 12.9 | 17.23 | 11.20 | 0.0 |
| Parlier, CA | 36.60 | 26 Feb 2024 | 19.5 | 7.1 | 12.6 | 12.83 | 11.24 | 0.0 |
| Parlier, CA | 36.60 | 27 Feb 2024 | 17.3 | 9.0 | 12.5 | 17.23 | 11.28 | 0.0 |
| Parlier, CA | 36.60 | 28 Feb 2024 | 20.0 | 6.1 | 11.9 | 17.40 | 11.31 | 0.0 |
| Parlier, CA | 36.60 | 29 Feb 2024 | 19.4 | 8.2 | 13.0 | 16.44 | 11.35 | 0.0 |

Tmax, Tmin and Tmean, daily maximum, minimum and mean air temperature; CDD, chilling degree-days. Fargo, ND. Data were obtained from the North Dakota Agricultural Weather Network (NDAWN) Fargo station (station no. 23; 46.89° N), retrieved 23 July 2026 from ndawn.ndsu.nodak.edu. Air temperature was reported in °F and total solar radiation in langleys, and was converted to °C as (°F − 32) × 5/9 and to MJ m⁻² d⁻¹ as Ly × 0.041868. The solar radiation value for 13 October 2023 is flagged by NDAWN as estimated. Parlier, CA. Station records from the California Irrigation Management Information System (CIMIS) station no. 39 (Parlier), at the University of California Kearney Research and Extension Center, were not accessible, and the values tabulated here were instead obtained from the ERA5/ERA5-Land reanalysis (Open-Meteo Historical Weather API) for the grid cell containing that site (36.59° N, 119.45° W, 104 m elevation), retrieved 23 July 2026. The variables used were daily temperature_2m_max, temperature_2m_min and temperature_2m_mean (°C) and shortwave_radiation_sum (MJ m⁻² d⁻¹). These are modelled reanalysis values rather than station observations and are interpreted accordingly; the Parlier record is used to characterize the mild-winter environment in general terms and not to derive any parameter of the models reported in the main text. Day length was computed astronomically from latitude and day of year using the CBM model (Forsythe et al., 1995, Ecological Modelling 80: 87–95) with a daylight coefficient of 0.8333°, corresponding to sunrise and sunset at the upper limb of the sun. It is a calculated rather than a measured quantity. CDD is the daily chilling degree-day accumulation on a 5 °C base, max(0, 5 − Tmean), as used for the accumulated-chilling axis of the Weibull model and for the chilling signal χ(t) of the state-space model (Section 2.5). Conditions in the greenhouse were imposed rather than observed and are given separately in Supplementary Table S3.

**Table S3.** Week-by-week photoperiod and temperature schedule of the greenhouse short-day and cooling regime.

| **Week** | **Timing** | **Photoperiod (light : dark, h)** | **Day temperature (°C)** | **Night temperature (°C)** | **Notes** |
| --- | --- | --- | --- | --- | --- |
| Pre-treatment | Start − 7 d | 16 : 8 | 25 | 20 | Conditions before the regime began |
| Week 1 | Start + 0 d | 15 : 9 | 25 | 20 |  |
| Week 2 | Start + 7 d | 14 : 10 | 25 | 20 |  |
| Week 3 | Start + 14 d | 13 : 11 | 23 | 18 |  |
| Week 4 | Start + 21 d | 12 : 12 | 23 | 18 |  |
| Week 5 | Start + 28 d | 11 : 13 | 23 | 18 |  |
| Week 6 | Start + 35 d | 10 : 14 | 21 | 16 |  |
| Week 7 | Start + 42 d | 9 : 15 | 21 | 16 |  |
| Week 8 | Start + 49 d | 8 : 16 | 21 | 16 | End of regime; vines transferred to 4 °C |
| 4 °C hold | Start + 56 d | 8 : 16 | 4 | 4 | Constant 4 °C under an 8-h/16-h photoperiod |
| G-1 | Start + 77 d | 8 : 16 | 4 | 4 | First DTA, after 3 weeks at 4 °C |
| G-2 | Start + 84 d | 8 : 16 | 4 | 4 | Second DTA, after 4 weeks at 4 °C |

DTA, differential thermal analysis. Timing is expressed relative to the start of the regime, which was initiated once vines had reached 12 nodes (Section 2.2); the corresponding calendar dates are given in Table S1. The photoperiod was shortened by 1 h per week from 16 h to 8 h over eight weeks and the temperature was lowered in 2 °C steps from 25/20 °C (day/night) to 21/16 °C, a total decline of 4 °C, as described in Section 2.2. The total decline and the step size are as specified in the Methods; the distribution of the two steps across the eight weeks is shown as applied. At the end of week 8 the vines were transferred to a 4 °C chamber under an 8-h/16-h photoperiod, and DTA was performed after 3 weeks (G-1) and 4 weeks (G-2). The regime combined a shortening photoperiod with a moderate temperature decline followed by a constant 4 °C hold, and at no point included exposure below 4 °C. It is therefore a short-day and moderate-cooling treatment rather than a photoperiod-only treatment, and the hardiness attained under it is interpreted throughout as the hardiness achievable without sub-zero exposure.

**Table S4.** Sensitivity of the chilling-based models to the choice of base temperature for chilling degree-day accumulation.

| **Base temperature (°C)** | **λ (CDD)** | **κ** | **τ₅₀ (CDD)** | **R² (Weibull)** | **aP** | **aC** | **d** | **R² (state-space)** | **Chilling contribution (°C)** | **ρ vs 5 °C** |
| --- | --- | --- | --- | --- | --- | --- | --- | --- | --- | --- |
| 0 | 261 | 0.24 | 57 | — | 0.11 | 1.49 | 0.45 | — | 7.9 | 0.965 |
| 5 | 273 | 0.93 | 184 | 0.75 | 0.11 | 1.00 | 0.45 | 0.57 | 6.7 | 1.000 |
| 10 | 467 | 0.33 | 153 | — | 0.11 | 0.92 | 0.45 | — | 7.9 | 0.971 |

CDD, chilling degree-days; λ, Weibull scale parameter; κ, Weibull shape parameter; τ₅₀, half-hardening point; aP, photoperiod gain; aC, chilling gain; d, deacclimation coefficient; ρ, Spearman rank correlation. The base temperature governs both the accumulated-chilling axis of the Weibull model and the chilling signal χ(t) of the state-space model (Section 2.5). A base of 5 °C was adopted in the main analysis for consistency with the thermal-time model of Ferguson et al. (2011); that row is shown in bold and reproduces the values reported in Table 2. For the 0 °C and 10 °C bases the chilling axis was rescaled in proportion to the daily chilling accumulated at that base and the Weibull function was refitted; aC was rescaled by the corresponding change in the mean chilling signal, with aP and d held at their calibrated values. Goodness-of-fit statistics are reported only for the calibrated 5 °C model, since the refitted models are not independently calibrated. λ and τ₅₀ are expressed in chilling degree-days and therefore change with the base temperature by construction; their absolute values are not comparable between rows and should be read relative to the total chilling accumulated at the same base. ρ is the Spearman rank correlation between the genotype-level τ₅₀ estimates obtained at the given base and those obtained at 5 °C. Values of 0.97 and above indicate that the ranking of genotypes by hardening kinetics is essentially unaffected by the choice of base temperature. The estimated contribution of sustained sub-zero chilling ranges from 6.7 to 7.9 °C across the three bases, so the conclusion that chilling contributes approximately 6–8 °C of hardiness beyond that attained under the greenhouse regime does not depend on this choice.

**Table S5.** Modelled loss of acclimated bud hardiness during simulated midwinter warm spells of three durations, for the 53 F1 genotypes.

| **Genotype** | **PRI** | **DRI** | **Peak field H (°C)** | **Greenhouse H (°C)** | **Loss, 5 d (%)** | **Loss, 10 d (%)** | **Loss, 15 d (%)** | **Hardiness retained after 10 d (%)** |
| --- | --- | --- | --- | --- | --- | --- | --- | --- |
| **536-078** | **0.83** | **0.17** | **27.1** | **24.6** | **5.5** | **9.3** | **11.9** | **90.7** |
| **539-127** | **0.79** | **0.21** | **23.3** | **21.1** | **6.6** | **11.1** | **14.2** | **88.9** |
| **536-142** | **0.70** | **0.30** | **21.6** | **19.0** | **9.6** | **16.1** | **20.6** | **83.9** |
| 539-013 | 0.58 | 0.42 | 20.7 | 17.5 | 13.1 | 22.1 | 28.3 | 77.9 |
| 539-041 | 0.55 | 0.45 | 22.8 | 18.4 | 14.4 | 24.2 | 30.9 | 75.8 |
| 536-175 | 0.54 | 0.46 | 24.9 | 19.4 | 14.4 | 24.3 | 31.0 | 75.7 |
| 536-157 | 0.45 | 0.55 | 22.1 | 17.1 | 17.6 | 29.6 | 37.8 | 70.4 |
| 539-073 | 0.43 | 0.57 | 23.0 | 17.3 | 18.0 | 30.4 | 38.8 | 69.6 |
| 536-019 | 0.41 | 0.59 | 22.0 | 16.7 | 18.6 | 31.4 | 40.1 | 68.6 |
| 536-005 | 0.41 | 0.59 | 22.8 | 16.9 | 18.8 | 31.7 | 40.5 | 68.3 |
| 539-162 | 0.36 | 0.64 | 24.5 | 17.1 | 20.4 | 34.3 | 43.8 | 65.7 |
| 539-126 | 0.34 | 0.66 | 23.1 | 16.4 | 21.0 | 35.3 | 45.1 | 64.7 |
| 536-009 | 0.33 | 0.67 | 23.4 | 16.4 | 21.1 | 35.5 | 45.4 | 64.5 |
| 539-026 | 0.33 | 0.67 | 25.5 | 17.1 | 21.1 | 35.6 | 45.5 | 64.4 |
| 539-010 | 0.33 | 0.67 | 19.4 | 15.1 | 20.9 | 35.2 | 45.0 | 64.8 |
| 536-043 | 0.31 | 0.69 | 22.4 | 15.9 | 21.8 | 36.7 | 46.9 | 63.3 |
| 536-182 | 0.27 | 0.73 | 22.7 | 15.7 | 23.0 | 38.7 | 49.5 | 61.3 |
| 536-023 | 0.26 | 0.74 | 24.9 | 16.1 | 23.3 | 39.2 | 50.0 | 60.8 |
| 536-134 | 0.25 | 0.75 | 23.1 | 15.6 | 23.6 | 39.8 | 50.8 | 60.2 |
| 536-117 | 0.23 | 0.77 | 25.9 | 16.0 | 24.3 | 40.9 | 52.2 | 59.1 |
| 536-064 | 0.23 | 0.77 | 23.7 | 15.4 | 24.4 | 41.1 | 52.5 | 58.9 |
| 539-069 | 0.23 | 0.77 | 23.5 | 15.4 | 24.4 | 41.1 | 52.5 | 58.9 |
| 539-104 | 0.22 | 0.78 | 25.5 | 15.7 | 24.8 | 41.8 | 53.4 | 58.2 |
| 536-073 | 0.20 | 0.80 | 23.7 | 15.1 | 25.2 | 42.5 | 54.3 | 57.5 |
| 539-044 | 0.17 | 0.83 | 24.1 | 14.9 | 25.5 | 42.9 | 54.8 | 57.1 |
| 539-125 | 0.17 | 0.83 | 22.5 | 14.6 | 24.4 | 41.1 | 52.5 | 58.9 |
| 536-008 | 0.16 | 0.84 | 21.8 | 14.4 | 23.8 | 40.1 | 51.3 | 59.9 |
| 536-055 | 0.15 | 0.85 | 20.9 | 14.2 | 22.9 | 38.5 | 49.2 | 61.5 |
| 536-180 | 0.14 | 0.86 | 22.2 | 14.3 | 24.2 | 40.7 | 52.0 | 59.3 |
| 539-119 | 0.10 | 0.90 | 24.6 | 14.2 | 25.7 | 43.3 | 55.3 | 56.7 |
| 539-153 | 0.10 | 0.90 | 24.6 | 14.1 | 25.7 | 43.3 | 55.3 | 56.7 |
| 539-072 | 0.09 | 0.91 | 24.0 | 14.0 | 25.4 | 42.7 | 54.6 | 57.3 |
| 539-085 | 0.08 | 0.92 | 23.3 | 13.8 | 24.9 | 42.0 | 53.7 | 58.0 |
| 539-089 | 0.03 | 0.97 | 22.3 | 13.3 | 24.2 | 40.8 | 52.1 | 59.2 |
| 539-088 | 0.01 | 0.99 | 24.9 | 13.1 | 25.8 | 43.5 | 55.6 | 56.5 |
| 539-151 | 0.00 | 1.00 | 24.4 | 13.1 | 25.6 | 43.2 | 55.2 | 56.8 |
| 539-067 | 0.00 | 1.00 | 23.1 | 10.4 | 24.8 | 41.8 | 53.4 | 58.2 |
| 536-169 | 0.00 | 1.00 | 23.3 | 12.3 | 25.0 | 42.0 | 53.7 | 58.0 |
| 536-067 | 0.00 | 1.00 | 23.4 | 10.8 | 25.0 | 42.2 | 53.9 | 57.8 |
| 536-124 | 0.00 | 1.00 | 19.4 | 10.6 | 20.8 | 35.1 | 44.8 | 64.9 |
| 536-002 | 0.00 | 1.00 | 23.9 | 12.8 | 25.4 | 42.7 | 54.6 | 57.3 |
| 539-043 | 0.00 | 1.00 | 16.3 | 10.1 | 10.5 | 17.7 | 22.6 | 82.3 |
| 536-186 | 0.00 | 1.00 | 15.3 | 9.1 | 1.8 | 3.0 | 3.9 | 97.0 |
| 536-198 | 0.00 | 1.00 | 23.6 | 12.9 | 25.2 | 42.4 | 54.1 | 57.6 |
| 536-179 | 0.00 | 1.00 | 24.5 | 12.4 | 25.7 | 43.3 | 55.3 | 56.7 |
| 539-101 | 0.00 | 1.00 | 22.9 | 12.0 | 24.7 | 41.7 | 53.2 | 58.3 |
| 539-108 | 0.00 | 1.00 | 20.2 | 11.6 | 22.1 | 37.2 | 47.6 | 62.8 |
| 539-079 | 0.00 | 1.00 | 22.5 | 12.2 | 24.5 | 41.2 | 52.6 | 58.8 |
| 539-142 | 0.00 | 1.00 | 14.7 | 9.4 | -8.7 | -14.7 | -18.8 | 114.7 |
| 539-170 | 0.00 | 1.00 | 22.6 | 11.0 | 24.5 | 41.2 | 52.6 | 58.8 |
| 539-171 | 0.00 | 1.00 | 24.5 | 13.0 | 25.6 | 43.2 | 55.2 | 56.8 |
| 539-176 | 0.00 | 1.00 | 21.0 | 13.0 | 23.1 | 38.9 | 49.7 | 61.1 |
| 539-185 | 0.00 | 1.00 | 21.6 | 12.1 | 23.7 | 39.9 | 51.0 | 60.1 |

PRI, Photoperiod Response Index; DRI, Deacclimation Risk Index; H, hardiness expressed as −LTE, so that larger values denote greater hardiness. Genotypes are ordered by DRI, from the least to the most chilling-dependent. The three genotypes with the lowest DRI (536-078, 539-127, 536-142) are shown in bold; these are the short-day and low-temperature responsive genotypes identified in Section 3.9. Peak field H is the mean of the two February field samplings and greenhouse H the mean of G-1 and G-2. PRI = (H_GH − H₀)/(H_Field − H₀) with H₀ = 13 °C, and DRI = 1 − PRI. The warm spell was simulated with the calibrated state-space model (aP = 0.11, aC = 1.00, d = 0.45) by setting the chilling signal χ(t) to zero, holding the photoperiod signal φ(t) at its 1 February value, raising the daily mean temperature to 10 °C, and integrating forward for 5, 10 or 15 days. Each genotype relaxes from its peak field hardiness toward the hardiness it sustains without sub-zero exposure, taken as its observed greenhouse value. Losses are expressed as a percentage of the acclimated hardiness gained above the deacclimated baseline, (H_peak − H₀), rather than in degrees, so that genotypes differing in absolute hardiness are comparable. Modelled losses are positively correlated with DRI (r = 0.44 for the 10-day scenario): the most chilling-dependent genotypes lose the largest fraction of their acclimated hardiness, whereas the three short-day and low-temperature responsive genotypes lose the least (9–16%). Genotype 539-142 shows a small negative modelled loss because its February field hardiness (14.7 °C) already lies below the level sustained by the model under thaw conditions; it is the most cold-susceptible genotype in the population and has essentially no acclimated hardiness to lose. These values are model projections and not observations. No warm spell was imposed experimentally, and the projections inherit all assumptions of the state-space model, including the definition of χ(t) and the assumed thaw profile. They are presented as a hypothesis about relative deacclimation risk that merits direct experimental testing.

**Table S6.** Genotype-level Weibull parameters with bootstrap 95% confidence intervals, fitted on the accumulated-chilling axis (base 5 °C).

| **Genotype** | **κ** | **95% CI for κ** | **CI width** | **λ (CDD)** | **95% CI for λ** | **τ₅₀ (CDD)** | **R²** |
| --- | --- | --- | --- | --- | --- | --- | --- |
| 536-157 | 0.10 | 0.10–0.18 | 0.08 | 239 | 151–355 | 7 | 0.55 |
| 536-073 | 0.13 | 0.10–0.22 | 0.12 | 14 | 5–42 | 1 | 0.57 |
| 539-176 | 0.29 | 0.12–0.47 | 0.35 | 591 | 332–3511 | 167 | 0.66 |
| 539-043 † | 0.30 | 0.21–1.00 | 0.79 | n.e. | n.e. | n.e. | n.e. |
| 539-108 | 0.40 | 0.10–1.17 | 1.07 | 495 | 197–5000 | 197 | 0.22 |
| 536-067 | 0.47 | 0.40–0.55 | 0.16 | 172 | 156–189 | 79 | 0.97 |
| 539-079 | 0.51 | 0.12–0.96 | 0.84 | 203 | 116–467 | 99 | 0.55 |
| 536-124 | 0.52 | 0.38–0.64 | 0.26 | 844 | 629–1384 | 417 | 0.92 |
| 536-186 † | 0.54 | 0.43–1.65 | 1.21 | n.e. | n.e. | n.e. | n.e. |
| 536-043 | 0.56 | 0.37–0.73 | 0.36 | 260 | 212–311 | 136 | 0.88 |
| 536-005 | 0.59 | 0.10–1.54 | 1.44 | 319 | 145–5000 | 171 | 0.30 |
| 536-179 | 0.63 | 0.28–1.05 | 0.77 | 162 | 108–229 | 90 | 0.71 |
| 539-073 | 0.63 | 0.22–1.08 | 0.86 | 239 | 166–980 | 133 | 0.62 |
| 539-010 | 0.63 | 0.19–1.88 | 1.68 | 619 | 339–5000 | 345 | 0.40 |
| 539-126 | 0.64 | 0.10–1.97 | 1.87 | 171 | 48–342 | 96 | 0.43 |
| 539-142 † | 0.67 | 0.58–1.25 | 0.66 | n.e. | n.e. | n.e. | n.e. |
| 539-171 | 0.70 | 0.56–0.85 | 0.29 | 142 | 127–160 | 84 | 0.95 |
| 536-198 | 0.71 | 0.26–1.23 | 0.97 | 168 | 116–261 | 101 | 0.69 |
| 536-002 | 0.74 | 0.44–1.08 | 0.63 | 207 | 161–270 | 126 | 0.82 |
| 539-067 | 0.87 | 0.78–0.98 | 0.20 | 255 | 238–274 | 167 | 0.99 |
| 539-153 | 0.94 | 0.73–1.20 | 0.47 | 170 | 154–195 | 115 | 0.95 |
| 539-101 | 0.95 | 0.59–1.37 | 0.78 | 243 | 202–343 | 165 | 0.87 |
| 536-008 | 0.97 | 0.53–1.51 | 0.99 | 349 | 262–546 | 240 | 0.79 |
| 539-119 | 0.99 | 0.42–2.58 | 2.16 | 137 | 94–215 | 94 | 0.69 |
| 539-041 | 1.00 | 0.70–1.38 | 0.68 | 258 | 223–340 | 179 | 0.91 |
| 536-142 | 1.02 | 0.73–1.38 | 0.65 | 352 | 291–441 | 245 | 0.89 |
| 539-170 | 1.07 | 1.00–1.15 | 0.14 | 285 | 276–301 | 202 | 1.00 |
| 539-125 | 1.07 | 0.38–2.14 | 1.75 | 264 | 168–497 | 188 | 0.67 |
| 539-044 | 1.16 | 0.73–1.66 | 0.93 | 235 | 176–292 | 171 | 0.85 |
| 536-175 | 1.21 | 0.99–1.49 | 0.50 | 202 | 181–228 | 149 | 0.96 |
| 536-169 | 1.23 | 0.94–1.60 | 0.67 | 249 | 220–301 | 185 | 0.93 |
| 536-064 | 1.23 | 0.98–1.55 | 0.57 | 234 | 207–266 | 174 | 0.95 |
| 536-023 | 1.24 | 1.14–1.34 | 0.20 | 196 | 187–204 | 146 | 0.99 |
| 539-089 | 1.27 | 0.98–1.63 | 0.65 | 304 | 269–351 | 228 | 0.95 |
| 536-009 | 1.33 | 1.10–1.55 | 0.45 | 257 | 239–289 | 196 | 0.97 |
| 536-134 | 1.38 | 1.10–1.72 | 0.62 | 276 | 249–313 | 211 | 0.97 |
| 536-182 | 1.42 | 1.05–1.83 | 0.78 | 288 | 248–326 | 222 | 0.94 |
| 536-180 | 1.46 | 1.04–1.82 | 0.78 | 317 | 270–351 | 247 | 0.93 |
| 539-162 | 1.51 | 0.78–3.74 | 2.95 | 243 | 166–381 | 190 | 0.73 |
| 539-088 | 1.55 | 1.07–2.17 | 1.11 | 203 | 176–255 | 160 | 0.92 |
| 539-151 | 1.70 | 1.33–2.10 | 0.76 | 244 | 214–263 | 197 | 0.96 |
| 539-085 | 1.77 | 1.50–2.04 | 0.54 | 281 | 260–297 | 229 | 0.98 |
| 539-104 | 1.81 | 1.37–2.50 | 1.13 | 213 | 184–246 | 174 | 0.95 |
| 536-078 | 1.83 | 1.29–3.73 | 2.44 | 178 | 139–198 | 145 | 0.93 |
| 539-069 | 1.99 | 1.22–2.98 | 1.76 | 281 | 229–336 | 234 | 0.89 |
| 539-185 | 2.03 | 1.38–2.59 | 1.21 | 335 | 295–361 | 280 | 0.94 |
| 539-072 | 2.12 | 1.60–2.69 | 1.08 | 270 | 236–293 | 227 | 0.96 |
| 539-127 | 2.34 | 1.21–8.00 | 6.79 | 291 | 220–371 | 249 | 0.83 |
| 536-055 | 2.35 | 2.10–2.47 | 0.38 | 356 | 345–361 | 304 | 1.00 |
| 539-013 | 2.37 | 1.95–3.66 | 1.72 | 359 | 342–390 | 308 | 0.98 |
| 536-019 | 2.49 | 1.79–2.95 | 1.16 | 324 | 296–336 | 280 | 0.97 |
| 539-026 | 2.96 | 1.95–4.18 | 2.23 | 101 | 97–116 | 89 | 0.87 |
| 536-117 | 3.06 | 2.40–4.27 | 1.86 | 116 | 113–126 | 103 | 0.95 |

CDD, chilling degree-days; κ, Weibull shape parameter; λ, Weibull scale parameter; τ₅₀ = λ(ln 2)^(1/κ), the half-hardening point; CI, confidence interval; n.e., not estimable. Each fit uses the five NDSU field sampling points of the genotype concerned, with Hmin and Hmax fixed at the population values of 13 and 27 °C so that shape parameters are comparable across genotypes, and λ and κ estimated by non-linear least squares. Confidence intervals are percentile intervals from 600 non-parametric bootstrap resamples of the residuals. † For 3 genotypes (539-043, 536-186, 539-142) the fit did not converge to an interior solution: the scale parameter reached the upper bound of the search interval and the fitted curve did not improve on the genotype mean, giving a negative coefficient of determination. These three genotypes are among the most cold-susceptible in the population and showed little or no increase in hardiness across the field sampling window, so the accumulated-chilling axis carries no hardening signal for them and λ, τ₅₀ and R² are not estimable. Their κ values are reported for completeness but should not be interpreted. Genotypes are ordered by κ. Across the 50 genotypes with a converged fit the median shape parameter is κ = 1.11, and the median across the full set of 53 genotypes is κ = 1.07; individual estimates range from 0.10 to 3.06. Genotypes with κ < 1 show a rapid initial response that decelerates, whereas genotypes with κ > 1 show a delayed, sigmoidal response. The mean width of the 95% confidence interval on κ is 1.05 and the interval includes κ = 1 for 24 of the 53 genotypes (45%). Genotype-level shape parameters are therefore only weakly identified from five sampling points. The distribution of κ should accordingly be read as evidence that genotypes differ in hardening kinetics rather than as a set of reliable individual estimates, and this limitation is stated in Section 4.4. Denser sampling through the acclimation window would be required to estimate genotype-specific hardening kinetics with confidence.

**Figure S1.** Daily air temperature and day length at the two outdoor sites through the 2023–2024 acclimation window. Upper panel: daily maximum, minimum and mean air temperature at the NDSU field site (Fargo, ND) and at the California vineyard site (Parlier, CA), from 1 September 2023 to 29 February 2024. Lower panel: astronomical day length at the two latitudes over the same period. Vertical lines mark the nine sampling times listed in Table S1. Temperature data are those tabulated in Table S2 and were obtained from the weather stations identified there. Day length was computed astronomically from latitude and day of year. Conditions in the greenhouse were imposed rather than observed and are not shown; they are given in Table S3.
